# The Decay of Motor Memories Is Independent of Context Change Detection

**DOI:** 10.1371/journal.pcbi.1004278

**Published:** 2015-06-25

**Authors:** Andrew E. Brennan, Maurice A. Smith

**Affiliations:** 1 School of Engineering and Applied Sciences, Harvard University, Cambridge, Massachusetts, United States of America; 2 Center for Brain Science, Harvard University, Cambridge, Massachusetts, United States of America; Johns Hopkins University, UNITED STATES

## Abstract

When the error signals that guide human motor learning are withheld following training, recently-learned motor memories systematically regress toward untrained performance. It has previously been hypothesized that this regression results from an intrinsic volatility in these memories, resulting in an inevitable decay in the absence of ongoing error signals. However, a recently-proposed alternative posits that even recently-acquired motor memories are intrinsically stable, decaying only if a change in context is detected. This new theory, the context-dependent decay hypothesis, makes two key predictions: (1) after error signals are withheld, decay onset should be systematically delayed until the context change is detected; and (2) manipulations that impair detection by masking context changes should result in prolonged delays in decay onset and reduced decay amplitude at any given time. Here we examine the decay of motor adaptation following the learning of novel environmental dynamics in order to carefully evaluate this hypothesis. To account for potential issues in previous work that supported the context-dependent decay hypothesis, we measured decay using a balanced and baseline-referenced experimental design that allowed for direct comparisons between analogous masked and unmasked context changes. Using both an unbiased variant of the previous decay onset analysis and a novel highly-powered group-level version of this analysis, we found no evidence for systematically delayed decay onset nor for the masked context change affecting decay amplitude or its onset time. We further show how previous estimates of decay onset latency can be substantially biased in the presence of noise, and even more so with correlated noise, explaining the discrepancy between the previous results and our findings. Our results suggest that the decay of motor memories is an intrinsic feature of error-based learning that does not depend on context change detection.

## Author Summary

Suppose you are asked to shoot free throws with a basketball. If you’re an unskilled shooter, you may at first miss in a consistent way for consecutive shots—perhaps a bit to the right—but you will soon learn to correct that error. However, an often-repeated finding is that if error information is withheld, such as if you close your eyes just after releasing the ball, performance will regress toward baseline. One explanation is that newly-formed motor memories are intrinsically volatile, decaying away if there is no continued performance feedback. However, recent work proposed an alternative mechanism: that newly-formed motor memories are intrinsically stable, but people change their behavior upon detecting a context change. This hypothesis predicts decay will occur only after the change is detected, leading to delayed decay if the context change is purposefully masked in an experiment. We show that previous estimates of decay onset delay, which provided support for the context-dependent decay hypothesis, were systematically biased and that decay instead begins immediately, without delay, even when context changes are effectively masked, in stark contrast to the 40+ trial delays previously reported. Thus, we show that recent memories decay independently of context change detection, suggesting that they are indeed intrinsically volatile.

## Introduction

Motor adaptation driven by performance error generally decays back toward baseline when error information is withheld or held near zero using zero-error clamp (zEC) trials [1–12]. This effect has conventionally been explained by the intrinsic decay hypothesis, which asserts that new learning is intrinsically volatile and decays partially on each subsequent trial. Perhaps the most direct evidence for decay in the absence of error comes from Scheidt et al. [1], who compared the decay of force field (FF) adaptation when error was clamped at zero against “washout” when the FF was removed and no external forces were applied. The zero-FF washout led to large aftereffect errors oppositely directed to the errors experienced during early training, resulting, unsurprisingly, in a rapid regression of learned adaptation toward baseline. In contrast, the zero error clamp (zEC) treatment eliminated performance errors but nevertheless resulted in a substantial, albeit protracted, regression of the learned adaptation toward baseline with about 50% decay occurring in the 150-trial decay period that was studied. Smith et al. [2] went on to show that two parallel adaptive processes decay at different rates during zEC trials. More recent work has suggested that decay can be driven separately by experience and by time [13], that experience-dependent decay displays local generalization [9], and that the asymptotic retention remaining after prolonged decay may be determined by reinforcement signals provided during training [8].

Recently, Vaswani & Shadmehr (henceforth V&S) [14] proposed context-dependent decay as an alternative to the intrinsic decay hypothesis. This hypothesis maintains that learning is intrinsically stable but intimately associated with the context in which it occurred, decaying only when a change of context is detected. This hypothesis makes two key predictions. First, it predicts that decay onset will be systematically delayed until a context change is detected. Second, it predicts that context changes that are more difficult to detect will result in prolonged delays and reduced decay amplitude at any given point of the retention period. V&S suggested that trial-to-trial kinematic variability is a key environmental feature that the motor system uses to detect changes in context and that many previous studies dramatically reduced the kinematic variability from the training period to the retention period by probing decay using zEC trials. Correspondingly, decay observed in these studies might result from the recognition of a context change based on the decreased kinematic variability inherent in zEC trial blocks compared to preceding FF trials. To probe decay in a manner that avoids this confound, V&S injected kinematic variability into EC blocks using variable error clamp (vEC) trials whose direction varied subtly from one trial to the next in a manner that matched the kinematic variability of late FF training, thereby minimizing the context change between the FF training and EC retention blocks. They reported that using vEC trials in place of zEC trials in the retention period resulted in dramatic reductions in the amount of decay and profound increases in the delay before decay onset, suggesting that variability-driven context change detection, rather than intrinsic decay, may be responsible for the decay of motor memories.

Given the radically different nature of this new hypothesis and the importance of understanding the mechanisms by which motor learning decays, further examination is crucial. In particular, there were technical issues with both the evidence for delayed decay onset and the evidence for the reduction in decay amplitude in the vEC condition in the V&S study. First, the reported decay onset latencies resulted from a parameter estimation procedure likely to be biased because of the constraints that were imposed. Second, the reduced decay reported for vEC-based retention was observed in an unbalanced experimental design without reference to baseline performance and without comparison to the analogous zEC-based condition. Thus, here we reexamine the context-dependent decay hypothesis using a balanced experimental design that references baseline performance and compares analogous vEC and zEC conditions. We then estimate decay onset latency using an unbiased variant of the previous individual-level analysis as well as a more powerful group-level approach. The results show no evidence for systematically delayed decay in any experimental condition and that reducing the detectability of the context change using vEC trials has essentially no effect on either decay onset latency or the amount of decay.

## Results

We investigated whether the detection of a context change underlies the decay of motor adaptation, first by manipulating the salience of the transition from training to retention periods, and second by examining whether a latent period occurs prior to decay onset corresponding to the time before context changes are detected.

**Fig 1:**
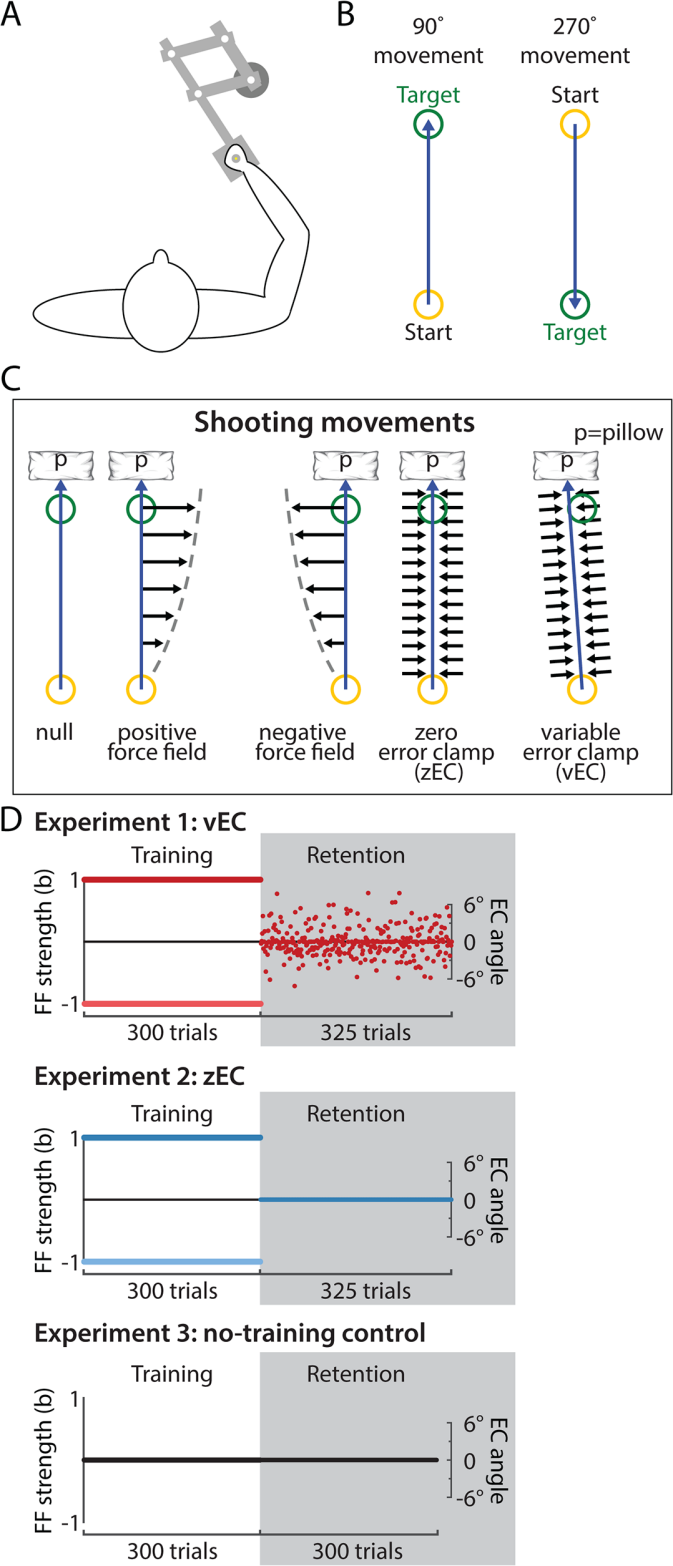
Experimental paradigm. (A-B) Participants grasped the handle of a 2-link robotic manipulandum to make movements in one of two directions. All shooting movements were performed in the 90° direction. (C) Shooting movements were to be aimed at the target but “shot” through it and brought to rest by a virtual pillow (p) created by the robotic arm 1cm beyond the target center. Experiments had a training block consisting of positive force field (+FF) trials, negative FF (−FF) trials, or null trials (0-FF) in which the robotic manipulandum applied forces (black horizontal arrows) proportional to the movement velocity and directed orthogonally to the movement direction. This training block was followed by a retention block of error clamp trials, where forces were applied reactively with a virtual channel in order to effectively constrain motion to a predefined straight-line path. Zero-error clamp (zEC) retention trials were always directed toward the target’s center, resulting in very low directional variability. In contrast, variable error clamp (vEC) trials were directed along a different non-zero angle on each trial and were used to impose subtle directional variations (σ = 2.6°) from one trial to the next during the retention period. The amount of directional variability in the vEC trials was matched to the directional variability late in FF training, thereby reducing the context change from the training environment. Point-to-point movements were performed analogously but stopped at the target as illustrated in the supporting information (S1 File). (D) Each experiment began with a training period of FF trials. For experiments 1 and 2, there were two subgroups (dark and light colors), one training on +FF and the other training on—FF trials; both subgroups had the same retention periods. Experiment 1 had a vEC-based retention period, and had two variants: experiment 1a (n = 20, 10 on +FF and 10 on—FF) in which all subjects had the same pre-selected sequence of errors in the retention period; and experiment 1b (n = 20, 10 on +FF and 10 on—FF) in which all subjects had the mirror-opposite sequence of errors in the retention period. Experiments 2 and 3 had retention periods based on zEC trials. Note that the force field strength (b) was controlled during the training blocks while the error clamp angle was controlled during the retention blocks.

In order to minimize the context change following a 300-trial force field training period, we used directionally variable error clamp (vEC) trials in place of zero-error clamp (zEC) trials during a 325-trial retention period, as first suggested by V&S. Each vEC trial clamped the direction of movement to a prespecified angle, and this angle varied subtly from one trial to the next in a manner that matched the movement direction variability experienced late in the training period (Fig 1C and 1D). This masked the context change between training and retention periods by allowing movement direction variability to be maintained rather than be dramatically reduced as it would be during zEC-based retention.

### Variable error clamps reduce the salience of context changes in a retention block

**Fig 2:**
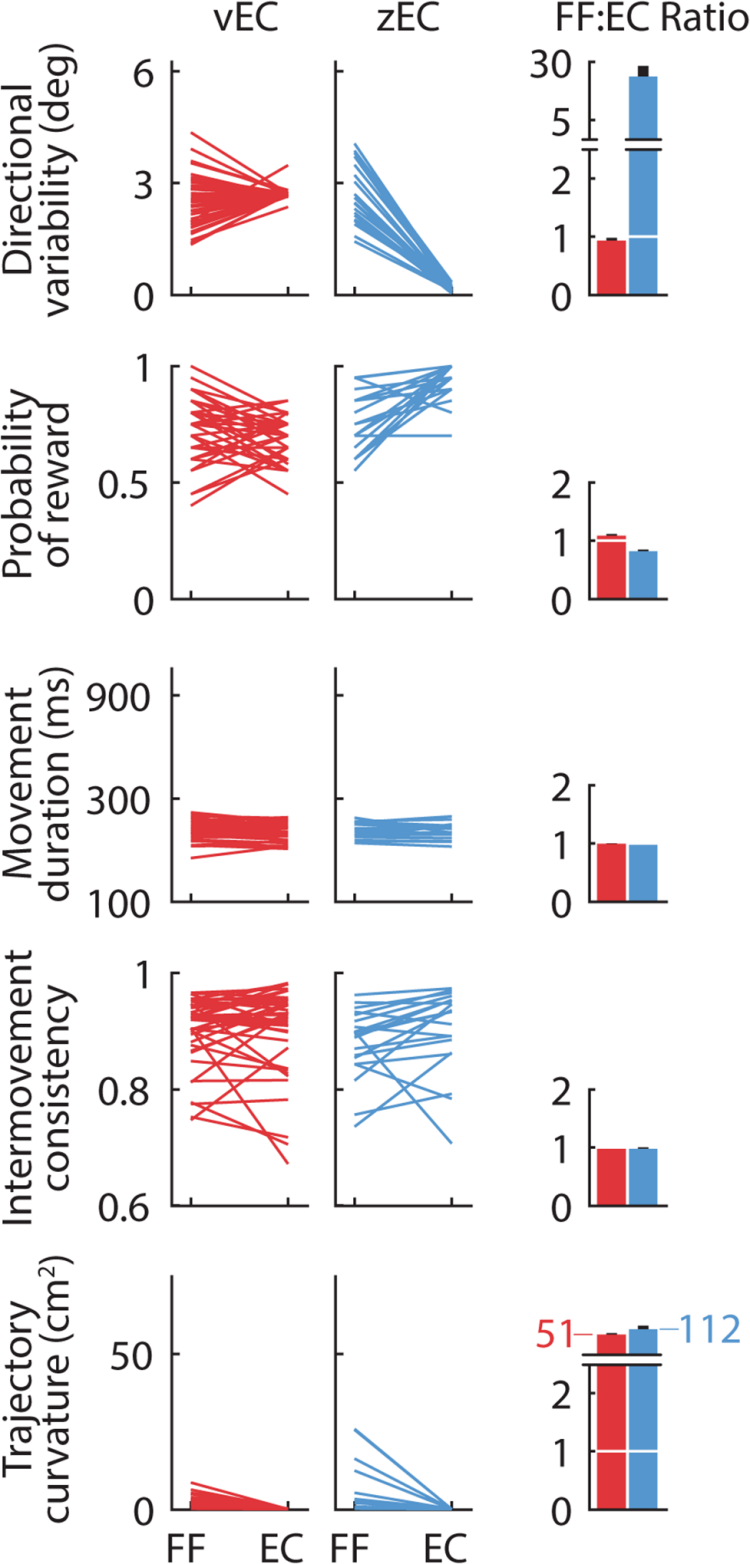
Comparison of movement characteristics during late training and early retention trials. Lines connect the average values for the last 20 training trials (FF) and the first 20 retention trials (EC) for each subject in the shooting movement experiments for the 5 movement characteristics that Vaswani and Shadmehr ([14], V&S) used: *Directional Variability* (Endpoint Standard Deviation in V&S) is the standard deviation of movement angle; *Probability of Reward* is the observed reward frequency; *Movement Duration* is the time to the target; *Intermovement Consistency* measures the similarity of consecutive movements [15]; *Trajectory Curvature* (Trajectory Deviation in V&S) measures the curvature of the movement, and is the sum of squared lateral deviations from the straight path joining the start and end positions of that path. Subjects could use large differences in these characteristics between the training and retention blocks, as quantified by the ratio of the last 20 training trials to the first 20 retention trials (rightmost column), to detect context changes between these blocks. For all five statistics, the vEC retention blocks better match the statistics of the training environment than their zEC analogs, suggesting that the vEC context change should be harder to detect. The values and ratios we observe are very similar to those reported in V&S. Error bars show SEM.

Experiments 1 and 2 compare vEC- and zEC-based retention for shooting movements, which V&S studied. We instituted the vEC manipulation in experiment 1 by choosing the EC direction on each trial from a Gaussian distribution with a mean of 0° and a standard deviation of 2.6°, replicating the magnitude of directional variability used in V&S. As a result, the directional variability we observed in the vEC retention period far exceeded that in the zEC retention period (2.70°±0.14° for vEC vs 0.16°±0.08° for zEC, mean±sd), but closely matched that from late in the training period in our data (2.52°±0.69°) (Fig 2 top row). Compared to the zEC retention period in experiment 2, the vEC retention period in experiment 1 also resulted in a reward frequency that better matched the late training period and a moderately stabilized trajectory curvature (Fig 2). Inter-movement consistency, which measured the similarity of consecutive movements [15], and movement duration did not change systematically from training to retention trials in either experiment (Fig 2). Overall, the institution of a vEC-based retention period substantially reduced performance differences between the training and retention periods in our data, and to an extent comparable to V&S. In experiments 4 and 5 (S1 File), where we analogously compared vEC- and zEC-based retention for point-to-point movements, vEC-based retention was similarly effective at matching the features of late training movements (S2 File).

### Decay patterns are obscured by large systematic oscillations if vEC sequences are not balanced

**Fig 3:**
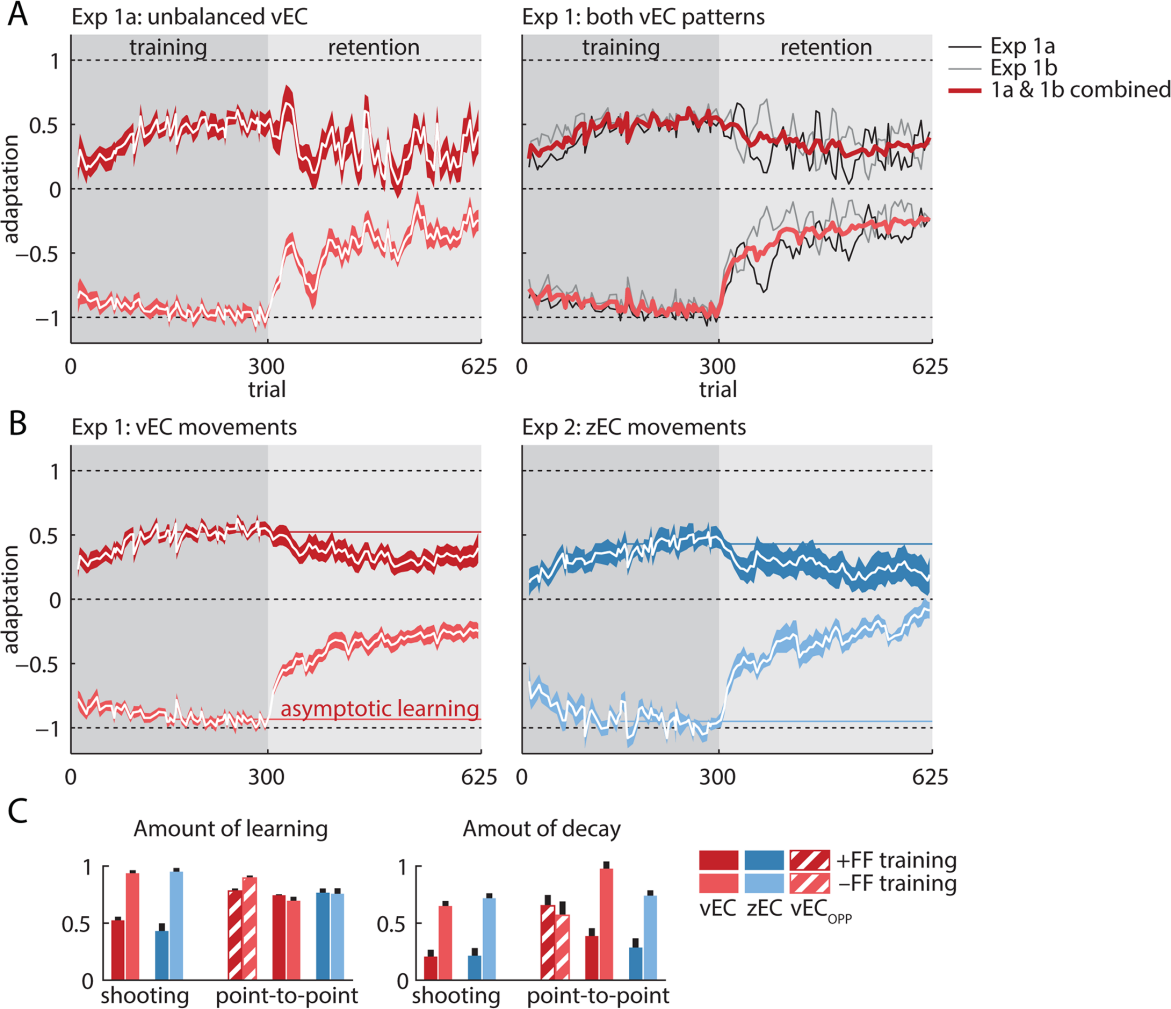
Raw learning and decay. (A) Left panel: motor adaptation and its decay during the training and retention periods in experiment 1a. There is clear adaptation, corresponding to the separation between the +FF and −FF groups (dark & light red) during training, and also clear decay, corresponding to the reduction of this separation during the retention period. However, there are large-amplitude oscillations in the retention period that obscure the underlying decay, especially if the +FF data are considered in isolation. Right panel: experiment 1b (mean shown in gray), which used the mirror-opposite vEC sequence, also shows large oscillations during the retention period but opposite in direction to the experiment 1a oscillations (mean shown in black). Combining the data from the two experiments (mean shown in red) balances the vEC sequence and largely eliminates the decay-obscuring oscillations, suggesting that they result from the specific vEC sequence employed. The vEC-balanced data reveal clear monotonic decay for both FF directions. (B) The learning and decay curves for the vEC-balanced experiment 1 data (red) and the zEC experiment 2 data (blue) closely match, suggesting little effect of context change salience on decay. In both cases, the +FF subgroups (darker colors) displayed highly attenuated learning and decay that was small but significant. In contrast, the −FF subgroups (lighter colors) displayed strong learning and robust decay. (C) Asymptotic learning was quantified using the average adaptation in the last 150 trials of the training block (100 trials for point-to-point zEC). Decay was quantified as the difference between asymptotic learning and the mean of all the retention trials after the first 150. These quantifications capture the asymmetries observed in the learning and decay curves (light versus dark bars), but more importantly show similar zEC and vEC learning (left panel) and decay (right panel), for both shooting and the point-to-point movements. For point-to-point movements, the hatched bars are experiment 4 (vEC_opp_) in the 90° direction, which is opposite to the zEC movement direction. The red and blue solid bars represent experiments 4 and 5 (vEC and zEC) in the 270° direction. Error bars show SEM.

A striking feature of the data from experiment 1a (n = 20) is the large irregular pattern of rapid trial-to-trial oscillations observed during the vEC-based retention period (Fig 3A, left), as in the V&S study. Unfortunately, these irregular oscillations largely obscure any pattern of decay that might be present during the retention period, especially for the +FF arm of the experiment for which the learning and decay amplitudes appears smaller than the −FF arm.

These retention period oscillations appear random in pattern, suggesting that they may simply reflect noisy results. However, these random-looking oscillations were surprisingly consistent across subjects, as evidenced by the tight error bars around them (Fig 3A, left). We hypothesized that these oscillations might be caused by the specific structure of the vEC sequence employed since, like V&S, experiment 1a used a single randomly-generated sequence of EC directions for all participants. In particular, the directional errors enforced by each vEC trial could lead to two types of systematic effects: same-trial motor responses due to limb stiffness and real-time feedback control, and next-trial responses due to motor adaptation. Together, these effects would result in a stereotyped time-varying pattern of motor responses specific to the particular vEC error sequence that could obscure the overall decay pattern.

We attempted to uncover the decay pattern by balancing out effects of the error sequence using a mirror-opposite vEC sequence in a second group of participants (experiment 1b, n = 20), which would be expected to cause the opposite stereotyped response. This mirrored vEC sequence injected the identical overall EC directional variability as experiment 1a and led to grossly similar learning and decay curves (Fig 3A, gray traces in the right panel). As expected, the retention period data from experiment 1b displayed large irregular oscillations like those observed in experiment 1a (Fig 3A, black traces in the right panel). But critically, the oscillations arising from this new vEC sequence almost perfectly mirrored those from the original sequence (Fig 3A, right). Thus, when we combined the data from the original vEC sequence in experiment 1a and the mirror-reversed sequence in experiment 1b, the oscillations largely cancelled out, evidenced by the trial-to-trial variance (see Methods) in the combined data being reduced by a remarkable 89% compared to experiment 1a. The vEC-balanced combined data thus display markedly cleaner retention curves. These cleaner retention curves reveal a clear pattern of decay for the vEC retention data in both the +FF and—FF subgroups and facilitate a more accurate comparison with the zEC data in experiment 2, which has no error-sequence-dependent oscillations because zEC trials do not induce an error (Fig 3B).

### Learning and decay appear asymmetric across FF directions but are unaffected by context change salience

When we compared the sequence-balanced vEC data from experiment 1 to the zEC data from experiment 2, we found a remarkable degree of similarity, at odds with the context-change detection hypothesis (Fig 3B). A 3-way analysis of variance (retention period type × FF direction × movement type) reveals that the amount of decay is not affected by the presence of vEC vs zEC-based retention periods (F_(1,76)_ = 0.13, p = 0.72), despite clear effects of positive versus negative FF directions (F_(1,76)_ = 109, p<10^–15^) and shooting vs point-to-point movements (F_(1,76)_ = 9.32, p = 0.003). Post hoc testing revealed significant decay in all the experimental subgroups (all 8 combinations of vEC vs zEC retention, +FF vs −FF training, and shooting vs point-to-point movements; p<0.02 in all cases). This indicates that vEC-based context change masking failed to prevent the decay of motor adaptation in our data.

In contrast to the striking similarity between the decay observed in vEC and zEC-based retention periods, we found that the positive and negative FF subgroups displayed markedly asymmetric learning and decay in both experiments 1 and 2 (Fig 3B and 3C). Asymptotic learning was nearly twice as large for the −FF compared to the +FF subgroups for both experiments 1 and 2 (0.52±0.03 for +FF vs 0.94±0.03 for −FF, p<10^–11^ in experiment 1 and 0.43 ±0.07 vs. 0.95±0.03, p<10^–5^ in experiment 2, mean±SEM). The amount of decay was also asymmetric, largely mirroring the asymmetric learning (0.21±0.06 vs 0.65±0.04, p<10^–6^, for the +FF vs −FF subgroups in experiment 1, and 0.21±0.07 vs 0.71±0.04, p<10^–4^ in experiment 2, Fig 3B and 3C). Whereas shooting movements in experiments 1–2 exhibited markedly asymmetric learning and correspondingly asymmetric decay across FF directions, analogous point-to-point movements in experiments 3–4 exhibited nearly symmetric learning (Fig 3C, left) but asymmetric decay (Fig 3C, right; learning and decay curves in S3 File). The marked asymmetries based on FF direction in this dataset stand in contrast to the similarity observed for decay in vEC vs zEC-based retention periods and underscores the importance of examining decay in an experimental design balanced for +FF and −FF training conditions to avoid the effects of selective sampling of the FF direction.

### The adaptation coefficient measure may not adequately characterize raw force profiles for shooting movements

Intriguingly, we found very similar small endpoint errors (both <1° in amplitude) during the training period for the +FF and −FF shooting movement subgroups (0.01±0.14° vs. 0.55±0.15° to the right), despite asymptotic adaptation that appeared to be nearly twice as high for the −FF condition. This, along with the fact that only shooting movements displayed asymmetric adaptation, led us to question whether shooting movement data were reasonably well characterized by regressing the force profiles onto the ideal force profile (the adaptation coefficient measure; see Methods), as we did in Fig 3 and V&S did throughout their study.

The adaptation coefficient measure is known to efficiently characterize adaptation for point-to-point movements, especially late in training, because the shapes of the learning-related change in force output closely match the ideal compensatory force [5,12,16,17] with correlations often above 90% [12]. Correspondingly, we find that experiments 4 and 5, which use point-to-point movements, result in force profiles whose shapes closely correspond to the ideal force pattern (R^2^ = 0.88, R^2^ = 0.88, and R^2^ = 0.94 for the subject-averaged +FF data and R^2^ = 0.78, R^2^ = 0.90 and R^2^ = 0.97 for the −FF data, for experiment 4 (90°), experiment 4 (270°), and experiment 5 (270°), respectively; S4 File panel A).

**Fig 4:**
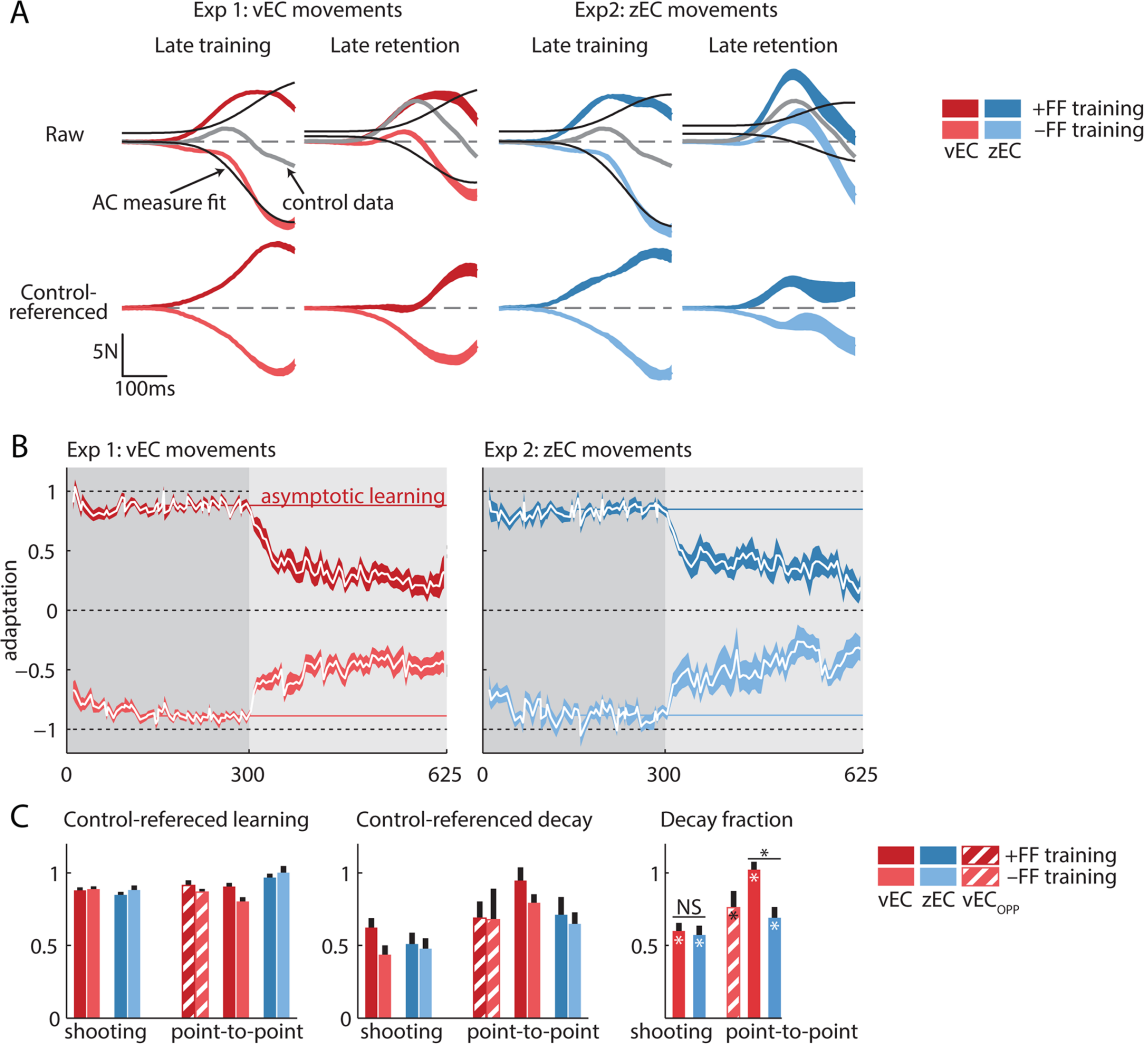
Learning and decay referenced to control data from a zero-FF training episode. (A) Top row: raw lateral force profiles for the +FF and −FF subgroups (darker and lighter colors, respectively, with experiment 1 in red, experiment 2 in blue, mean ± SEM). The +FF and −FF subgroups display differently-shaped force profiles but similar overall force levels during training, corresponding to similar average endpoint errors (not shown). The −FF subgroup data are well-captured by the adaptation coefficient measure (black line), which is a regression onto the ideal force profile, while the +FF data are not. This explains why learning and decay appear attenuated in the +FF subgroup in Fig 3. We performed a control experiment consisting of a 0-FF “training” block and a zEC retention block (experiment 3) to provide a baseline reference for adaptation and decay. Considering the force profiles (colored traces in top row) relative to this baseline reference (gray traces in top row) reveals symmetric adaptation and decay between +FF and −FF conditions for both training and retention (colored traces in second row). Note that the control experiment force profiles increase substantially during the retention period, indicating that the unreferenced shooting movement decay data in Fig 3 are confounded by a tendency to produce more positive force during an extended EC block. Without control-referencing, this tendency causes the +FF decay to be underestimated and the −FF decay to be overestimated, as it was in Fig 3. Since the control-referenced retention data is still not always well explained by the shape of the adaptation coefficient measure, we quantified adaptation using an integrated lateral force measure that is agnostic to the shape of the force profile. (B) Like Fig 3, the control-referenced vEC and zEC learning and decay appear similar (red vs blue), but here we also see symmetric learning and decay across +FF and −FF conditions in both experiments. The strong decay apparent in both the +FF and −FF arms of the vEC experiment is in contrast to reports of the vEC manipulation eliminating decay. (C) Consistent with the unreferenced adaptation coefficients in Fig 3, the analysis based on control-referenced integrated lateral forces shows similar learning and decay for the analogous vEC and zEC experiments, but it displays much greater symmetry across +FF vs −FF conditions. For point-to-point movements, the vEC condition (red) actually seems to increase the decay somewhat over the corresponding zEC data (blue). These data fail to support the prediction that vEC-based retention will reduce or eliminate decay. For point-to-point movements, the hatched bars are experiment 4 (vEC_opp_) in the 90° direction, which is opposite to the zEC movement direction. The red and blue solid bars represent experiments 4 and 5 (vEC and zEC) in the 270° direction. Error bars show SEM.

In contrast, the adaptation coefficient measure does not accurately characterize the +FF training data from experiments 1 and 2 as the force patterns observed during these shooting movements are systematically different from the shape of the ideal compensatory force pattern. This is evidenced by comparing the colored (observed force) and black (regression onto ideal force) curves in Fig 4A. Although shooting-movement force profile shapes closely correspond to the ideal force pattern for the −FF training data (lighter colors, R^2^ = 0.98 and R^2^ = 0.97 for experiments 1 and 2, respectively), the correspondence is dramatically reduced for the +FF training data (darker colors, R^2^ = 0.70 and R^2^ = 0.58 for experiments 1 and 2, respectively), resulting in lower adaptation coefficients. This poor correspondence becomes even worse for the +FF retention data (R^2^ = 0.55 and R^2^ = 0.14 for experiments 1 and 2, respectively).

### Control referencing reveals symmetric learning and decay but no effect of context change salience

Learning related changes in lateral force are generally assessed by subtracting out the force profiles measured during a baseline period [5,12,16,17] to isolate the learning-related changes from learning-unrelated performance biases. However, the experimental design employed in experiments 1 and 2, replicating the design in V&S, did not include a baseline period. We therefore performed a control experiment (experiment 3) consisting of a zero FF “training” period followed by a zEC “retention” period. This allowed us to provide not only a control reference for the training periods in the +FF and −FF arms of experiments 1–2, but also a control reference for the retention period data, which becomes important if error clamp exposure systematically changes the learning-unrelated performance biases. This control experiment enabled us to isolate changes in force output during both training and retention periods that specifically resulted from exposure to the training environment.

Remarkably, the asymmetry in raw force observed between the +FF and −FF shooting-movement conditions (Fig 4A, 1^st^ row) largely disappears when these data are considered relative to the control experiment by subtracting the mean force profiles from the control experiment (Fig 4A, 2^nd^ row). The control-referenced force profiles become highly symmetric across FF directions during both the late training and the late retention periods for both vEC and zEC experiments. This suggests that the raw force asymmetry we observe in the top row of Fig 4A arises largely from the lack of an appropriate reference. The increased symmetry for the control-referenced profiles compared to the raw force profiles is especially profound for the retention period data because continuous exposure to the error clamp trials in the retention period results in subtle but systematic changes in lateral force patterns, as evidenced by the difference in control experiment force profiles during late training and late retention (Fig 4A, top row, gray lines). These differences should be accounted for when evaluating the decay of a learned adaptation.

Despite the remarkable increase in symmetry, the shapes of the control-referenced force profiles still displayed some systematic differences from the ideal force profiles (black curves in the top row), indicating that the learning-related changes in these shooting movements may not be fully captured by the adaptation coefficient measure. We thus took a cautious approach of characterizing the force profiles associated with adaptation in a more general manner based on the total integrated lateral force, which is not sensitive to the shape of the force profile (see Methods). While this change of measure does further increase symmetry and more fully represents the data, we find very similar learning and decay using the control-referenced adaptation coefficient measure (S5 File).

Like the force profiles shown in Fig 4A, the learning and retention curves for shooting movements shown in Fig 4B are considerably more symmetric than their unreferenced counterparts shown in Fig 3B. Reanalyzing the data based on the integrated lateral force measure applied to control-referenced data reveals that the +FF and −FF groups display adaptation levels within 5% of one another during training (0.88±0.02(+FF) vs 0.89±0.02(−FF), p = 0.78 in experiment 1; 0.85±0.02 vs 0.88±0.03, p = 0.37 in experiment 2) and within 30% during retention (0.62±0.07 vs 0.44±0.06, p = 0.05 in experiment 1; 0.51±0.08 vs 0.48±0.07, p = 0.77 in experiment 2, Fig 4B), leading to highly symmetric decay amplitudes. The fraction of decay relative to the asymptotic adaptation level is similarly symmetric (73±8% vs. 51±8%, p = 0.07 for the +FF and −FF conditions in experiment 1, and 61±10% vs. 56±10%, p = 0.72 in experiment 2) (Fig 4C).

Similar to the findings in shooting movements, decay in the point-to-point movements also became substantially more symmetric when considered relative to an analogous control experiment while the previously-symmetric learning did not lose its symmetry (Fig 4C; learning curves in S4 File panel B).

While substantially changing the symmetry of the learning and decay curves, control-referencing and using integrated lateral force to quantify adaptation did not change the main findings from the analysis presented in Fig 3: vEC and zEC learning and decay curves appear very similar despite the vEC trials masking the context change. A 3-way analysis of variance (retention period type × FF direction × movement type) on the control-referenced data reveals decay (Fig 4C, middle panel) is not affected by the type of retention period (p>0.2), while the effects of FF direction and movement type are diminished relative to the unreferenced data, as expected due to the improved symmetry, although both remain significant factors (p = 0.04 and p<0.001, respectively). Correspondingly, the overall decay fraction, which combines the +FF and −FF control-referenced decay data from each experiment and normalizes it by the corresponding amount of learning, is similar for the vEC and zEC experiments (Fig 4C, right panel). In fact, the only statistical difference in overall decay fraction is in the point-to-point data where the 270° vEC experiment actually shows *more* decay on average than the zEC version (102±5% vs 71±7%, p<0.001).

Overall, the analyses performed in this section and illustrated in Fig 4 suggest that (1) omission of a baseline reference can skew measurements of adaptation and retention; (2) retention data are best referenced to error clamp data that follows a zero-training period since the learning-unrelated performance biases shift during this period; and (3) a regression-based adaptation coefficient measure may not adequately characterize the learning-related changes that occur for shooting movements. Incorporating these improvements increased the similarity between FF directions and retained the similarity between vEC and zEC retention periods.

We have thus found that, after performing both +FF and—FF training, balancing the vEC sequence (Fig 3), and control-referencing (Fig 4), there is a clear pattern of decay for both the vEC and zEC experiments, in both shooting and point-to-point movement paradigms (Fig 4C). Even more importantly, shooting movement vEC and zEC conditions result in remarkably similar learning and retention curves. We therefore conclude that, after accurately characterizing the adaptive changes in motor output induced by FF exposure, the reduced context change conferred by a vEC-based retention period does not reduce the decay of a trained adaptation. This finding contradicts the prediction of the context-dependent decay hypothesis.

### Estimating decay onset

To this point, we have found that the vEC and zEC retention periods induce similar decay amplitudes, at odds with the findings of V&S and the assertion that a masked context change would abolish decay. However, more direct predictions of context-dependent decay are the existence of a delay before decay onset and a dramatically extended delay when the context change is masked. We therefore closely examined decay onset time in vEC and zEC data.

Based on the findings from Fig 4, we used a control-referenced, integrated lateral force measure of the adaptive response to estimate decay onset latency. From Fig 3A, we knew that the individual data had vEC-sequence specific oscillations that increased the noise and would make the decay onset harder to estimate. In order to remove these oscillations, we first estimated the vEC-sequence effects as half the difference between the subject-averaged data from experiment 1a and that from experiment 1b, which had mirror-opposite vEC sequences. We then subtracted this estimate from each individual’s data. This reduced the trial-to-trial variance in the individual subject data by 49±3% and resulted in individual retention curves less contaminated by vEC-sequence specific oscillations.

We first examined decay onset using a group-level analysis that estimates the distribution of delays across subjects. We followed up with an analysis at the level of individual participants, where we fit delayed exponential functions to each participant’s adaptation curves, largely echoing the analysis presented in V&S.

### Group-level analysis reveals essentially no systematic delay in decay onset

The subject-averaged retention curves shown in Figs 3 and 4 all appear to begin decaying immediately after the retention period onset. However, the decay onset delay estimated from the mean retention curve could be much smaller than the average individual delay because a few early-decaying individuals would result in early decay in the mean data. Furthermore, a few early-decaying subjects are likely to exist, even when the mean delay is large, if the delays are exponentially distributed across individuals as V&S suggested. This is because an exponential distribution always has its mode at zero and thus results in a non-trivial number of near-zero samples. For example, even with a large mean delay of 90 trials, an exponential distribution of delays predicts 11% of participants to have delays below 10 trials, twice the number expected if the distribution was uniform. This would result in non-trivial nearly-immediate decay in the mean retention curve.

**Fig 5:**
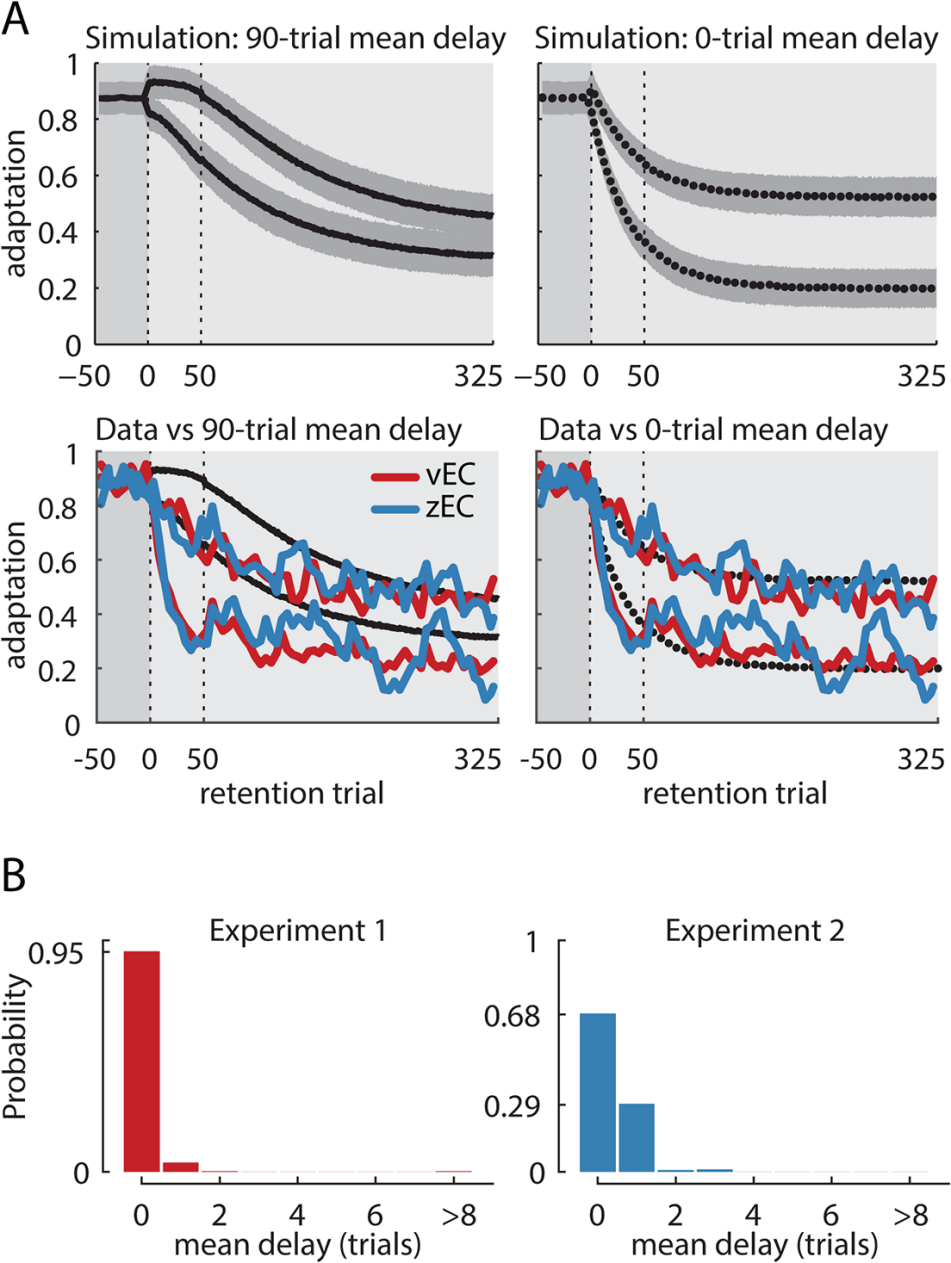
Group-level analysis of mean decay onset time. (A) Top row: Simulated decay for scenarios with subjects having an exponential distribution of delays with means of 90 and 0 trials. For each scenario, we simulated 40 subjects and median divided them into two equal groups based on their decay during the first 50 trials of the retention period. The black curves show the mean decay obtained from 1000 such simulations with shading corresponding to the standard deviation across simulations. The low-decay subgroups for the simulations with a 90-trial mean delay show no early decay, quantified as the ratio between decay in the first 50 vs the last 75 retention trials, and it even has slightly elevated average adaptation in the first 50 trials due to a selection bias (see Results). In contrast, both subgroups from the simulations with a 0-trial mean delay decay immediately. Second row: The data from experiments 1–2 were median-divided in the same way, based on the amount of decay during the first 50 trials. The means of each of these subgroups are plotted in red and blue for vEC and zEC, respectively. For both experiments, the experimental data appear consistent with the simulations for zero-delay but not 90-trial mean delay (dotted vs solid lines). Note that the simulated curves are smooth because they represent the mean of 1000 simulations, where individual simulations vary much like the data do. (B) Estimates of the mean delay in our data using a Bayesian inference procedure based on the early decay ratios. We used simulations to estimate the joint distribution P(ED_high_, ED_low_ | μ_λ_) for the mean delay μ_λ_ and the early decay ratios ED_high_ and ED_low_. This is a likelihood function for μ_λ_. We then assumed a uniform prior over the integer mean delays from 0 to 90 trials to estimate the posterior distribution P(μ_λ_ | ED_high_, ED_low_), which is proportional to P(ED_high_, ED_low_ | μ_λ_) ∙ P(μ_λ_) (see Methods). Large mean delays support the context detection hypothesis, which predicts decay to begin only after a change of context is detected. Near-zero delays are at odds with the context detection hypothesis because changes in variability cannot be detected immediately. The posterior distribution for experiments 1 (left) and 2 (right) are heavily skewed toward immediate decay, with a maximum *a posteriori* delay estimate of zero trials in both cases. The 95% confidence interval is 0 trials for experiment 1 and 0–1 trials for experiment 2, whose interval is somewhat more diffuse because it had 20 rather than 40 participants. These results are in stark contrast to the 90 and 40 trial mean delays reported for vEC and zEC shooting experiments in V&S, respectively.

We thus devised a method to estimate the average individual decay onset time from the group data in a way that was not sensitive to a fraction of individuals decaying early. To do this, we stratified subjects into two equal-sized groups according to the amount they decayed in the first 50 trials of the retention period—those who decayed most and those who decayed least (Fig 5B). If the distribution of decay onset delays was exponentially-shaped with a mean of 90 trials as V&S suggested, 57% of the subjects would display delays longer than 50 trials, so the small-decay subgroup should be composed largely or entirely of these individuals and would thus be expected to display nearly perfect retention during this 50-trial period. On the other hand, if decay onset was immediate, this small-decay subgroup would show rapid decay. We quantified the rapidity of decay onset using the ratio of early decay to late decay, which we call the early decay ratio.

We simulated this process using exponential population distributions of delays (i.e. each simulated subject was randomly assigned a delay drawn from an exponential distribution). We performed 1000 separate simulations for various mean delays, from 0 to 90 trials, with each simulation consisting of 40 subjects to match the number of subjects in experiment 1 (these simulations were repeated but with 20 subjects for each simulation as a reference for experiment 2, which had 20 participants). Each simulation also included population distributions for decay depth, decay rate, and measurement noise that were estimated from the data (see Methods). The 90-trial mean delay simulation (Fig 5A, left panel) resulted in a small-decay subgroup that displayed no apparent decay during the first 50 trials followed by a sharp decay thereafter. In fact, the simulation even showed slightly heightened adaptation during the first 50 trials due to a selection bias that occurred because there were many subjects without decay so those with the highest average random noise were chosen for the small-decay subgroup, resulting in a slightly negative early decay ratio (−0.12±0.08). In contrast, the large-decay subgroup rapidly decayed in the first 50 trials of the retention period (early decay ratio = 0.23±0.06). On the other hand, the simulation with a mean delay of 0 trials resulted in immediate decay for both subgroups following a brief bump caused by the noise selection bias (Fig 5A; early decay ratios of 0.35±0.06 and 0.50±0.03 for small and large decay groups, respectively). When we stratified the experimental data in the same way, we found that both experiment 1 vEC data and experiment 2 zEC data display immediate decay with patterns similar to those predicted by the simulation with a mean delay of 0 trials (immediate decay), but qualitatively different from those predicted by the simulation with a mean delay of 90 trials (Fig 5A and 5B). Note that the traces and shading for the simulations illustrate the means and standard deviations of the retention curve across 1000 simulations; individual simulations were much noisier, similar to the data. The corresponding early decay ratios were 0.31 and 0.67 for the small and big decay subgroups in experiment 1, and 0.40 and 0.69 in experiment 2. We also found the divisions to be balanced across FF direction and vEC sequence, with 10 (10) subjects in the low-decay group for experiment 1 coming from the +FF (–FF) direction and 9 (11) coming from experiment 1a (1b), while 4 (6) subjects in the low-decay subgroup for experiment 2 came from the +FF (–FF) direction, indicating that the median division was not selecting subjects based on some properties of the experimental manipulation but rather on their individual behavior.

These qualitative results indicate a mean delay much closer to 0 than the 90 trial estimate that V&S suggested, but do not provide rigorous statistical bounds on an estimate of the true mean delay before decay onset. To accomplish that, we used a Bayesian approach to estimate a probability distribution for the mean delay in our dataset, from which we could estimate confidence bounds. The context-dependent decay hypothesis posits an exponential distribution of delays over subjects with means as large as 90 trials for the vEC experiment. The intrinsic decay hypothesis posits immediate decay for all subjects, which implies an exponential distribution with mean 0 and can thus be considered part of the same family. We therefore framed the problem as determining confidence bounds on the mean delay from the family of exponential distributions with different means, with low mean delays favoring intrinsic decay and large mean delays favoring context detection.

Each of the 1000 simulations (above) yielded two scalars for each simulation: the early decay ratios for the high-decay and the low-decay median-divided subgroups. We characterized the joint distribution of these two values using a multivariate normal distribution parameterized by its mean and covariance. This yielded an estimate of the joint probability P(ED_large_, ED_small_ | μ_λ_) for the early decay ratios ED_large_ and ED_small_ given the mean delay μ_λ_, which is a likelihood function when viewed as a function of μ_λ_. Coupled with a uniform prior over the integer values from 0 to 90 trials for the mean delay μ_λ_, this likelihood function yielded the posterior distribution over the mean delays, given the data: P(μ_λ_ | ED_large_, ED_small_).

This analysis found maximum *a posteriori* estimates of mean delay to be zero trials for experiments 1 and 2. The 95% (99%) confidence intervals were the value 0 (interval [0 1]) for experiment 1, which had more data, and the interval [0 1] ([0 3]) for experiment 2, providing striking evidence against any substantially delayed decay and against any increases in delay when context changes are masked. These narrow confidence intervals reflect a profound change from the prior to the posterior, indicating that the observed early decay ratios provide strong evidence against a delayed decay onset.

### Fitting noisy data with delayed exponentials requires an unconstrained delay parameter

The 0-trial mean delay for vEC retention we found with 95% confidence using the group-level analysis stands in stark contrast to the 90-trial mean delay suggested by V&S based on an individual-level analysis. In order to understand the discrepancy between these results, we also performed an individual-level analysis to estimate decay onset delays in individual participants.

As with the group-level analysis, we first removed the vEC-induced oscillations from the individual subject data. However, as expected, the individual subject data were still quite noisy. This substantial noise causes statistical bias if the estimated decay onset is constrained to occur at or after the beginning of the retention block, as was done in V&S using the rationale that an earlier onset would require the subject to somehow anticipate the retention period and is therefore impossible. For noise-free data, such a constraint would not be problematic, but with any noise in the data, such a constraint would bias delay estimates toward the interior of the constraint region. This would have a particularly large effect if the true delay was zero, because positive-delay estimation errors would be accepted and negative-delay errors would not. This effect prevents unbiased hypothesis testing about whether decay onset is systematically delayed.

To estimate the importance of this effect, we repeatedly estimated the delay parameter, λ, from a simulated set of retention curves (n = 10,000). We added random white noise with an amplitude estimated from our shooting movement data (see Methods) to exponentially-decaying retention curves that began to decay immediately at retention period onset. Using a window of data spanning 150 trials before the retention period onset through 325 trials after it, we fit a delayed exponential to each noisy decay curve:

$$f(t)=\{\begin{array}{r} a,\;\text{if}\;t\leq \lambda \\ (a-b)\text{exp}\left(-\frac{\left(t-\lambda \right)}{\tau }\right)+b,\;\text{if}\;t>\lambda \end{array}$$

**Fig 6:**
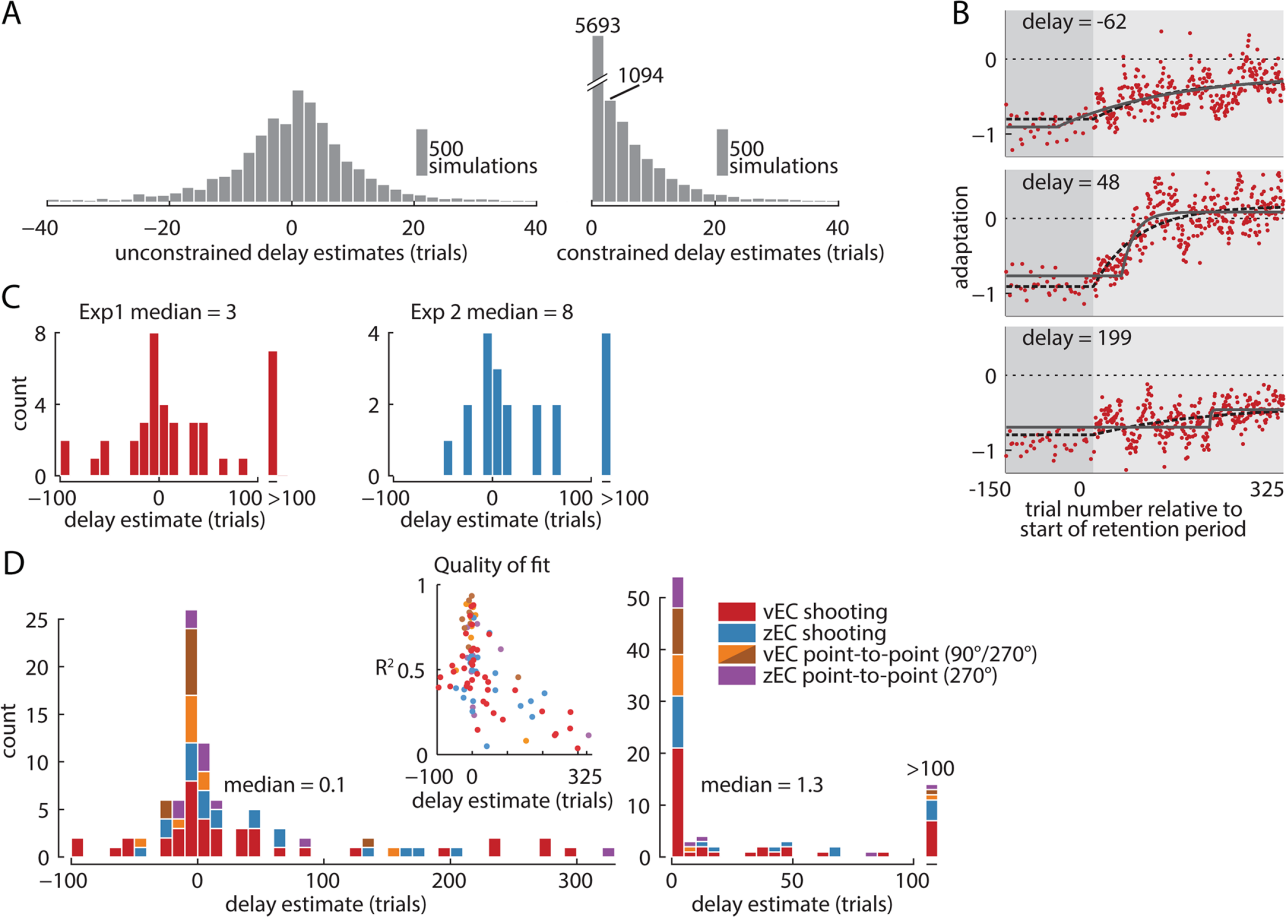
Individual-level analysis of decay onset time. (A) Distributions of delay parameter estimates obtained from fitting a delayed exponential to simulated noisy zero-delay data. Fits that did not constrain the delay parameter (left panel) resulted in a distribution of delay estimates centered near zero trials after the retention period onset with some positive and some negative delays. In contrast, constraining the fit delay to be non-negative (right panel) shifts the left half of the unconstrained distribution to zero while preserving the right half. Using the latter procedure, it impossible to meaningfully test for the existence of a delay since even the zero-delay simulation results in only positive values, substantially biasing the average delay estimate. (B) Three example subjects from experiment 1 with best-fit delayed exponentials (black lines). These subjects had best-fit delays that were moderately negative (top), moderately positive (middle), and highly positive (bottom). The dashed line represents the best-fit zero-delay exponential. (C) Delay parameter estimates for experiments 1 and 2 are centered near zero, with some positive and some negative estimated delays. The vEC condition (experiment 1) does not show more delay than the zEC condition (experiment 2), despite the masked context change that should make this change take longer to detect. (D) Histogram of delays aggregated across all experimental conditions based on unconstrained (left panel) and constrained (right panel) fits of the delay parameter. The right panel shows many positive delays but the potential negative delays that could balance these are not permitted by the fitting procedure so the significance of the positive delays cannot be discerned. In contrast, the left panel shows most delay estimates to be near zero, and is well-balanced between positive and negative values. The inset shows that the subjects who were well-fit by a delayed exponential tended to have delays near zero, while subjects who were poorly fit had delays more uniformly spaced throughout the fitting window [−100, 325], suggesting that the large amplitude delays likely arose from poor fitting rather than the existence of truly delayed decay behavior. These results are consistent with the group-level analysis.

We repeated the fitting both with and without constraining λ. Unconstrained fitting resulted in the expected bell-shaped distribution of λ estimates centered near zero. However, constraining λ≥0 resulted in λ estimates that were exclusively non-negative and with an approximately exponential-shaped distribution, similar to those reported in V&S (Fig 6A). This roughly exponential shape results from the combination of the positive half of the bell-shaped unconstrained distribution and a delta function at λ = 0 corresponding to the negative half of the unconstrained distribution—a combination that can appear to be exponentially distributed depending on the bin size.

Since the true delays were set to exactly zero in these simulations, both the impossible negative delays and the plausible positive delays from the unconstrained analysis could only result from the noise we injected corrupting the parameter estimates. In the unconstrained analysis, this noise systematically affects the spread of the fit values but not the mean, so we can test for the presence of a systematically delayed decay by examining the mean. In contrast, this noise in the constrained distribution systematically shifts the mean of the fit values, thus preventing the presence of any true delay in the data to be clearly identified or rejected. We therefore estimated λ without constraint.

### Individual-level analysis indicates essentially no systematic delay in decay onset

We examined the delay in the individual participant decay by fitting exponential functions with unconstrained delay parameters to the error clamp trials during the last 150 trials of the learning period (trials −150 to 0) and the entirety of the retention period (trials 1–325) (Fig 6B). The delay parameter, λ, represents the decay onset time for the best-fit delayed exponential function relative to the beginning of the retention period (see equation in the previous section).

In both experiments 1 and 2, we found the distributions of delays to be largely centered near zero (median = 3.4 (p = 0.10, Wilcoxon sign-rank test), and 7.6 (p = 0.04) trials, respectively, Fig 6C). These near-zero delays stand in contrast to the predictions of the context change detection hypothesis and the results of V&S that reported delays of 96±27 and 44±29 trials, respectively, for vEC and zEC-based retention. Moreover, we found the distributions of best-fit delays for experiments 1 and 2 to be very similar, despite the presence of a vEC manipulation that masked the context change in experiment 1. In fact, the shift in delays we observed was nominally in the wrong direction compared to the prediction of the context change detection hypothesis (p = 0.56, Mann-Whitney U-test). The point-to-point movements also displayed near-zero median delays with no significant differences between vEC and zEC-based retention (S6 File). Combining all the data to give a better picture of the full distribution of delay estimates highlights how most unconstrained delays are near zero, both positive and negative (Fig 6D). These results indicate neither systematically delayed decay onset nor an increase in the decay onset delay when the context change is more difficult to detect. This agrees with the group-level analysis presented above but is at odds with the context change hypothesis for decay.

In many ways, constraining delay values to be positive for the individual fits is analogous to applying a uniform prior over the positive mean delay values in the group-level analysis we performed above. Indeed, the individual-level constraint is equivalent to using a flat prior over the positive delays and using the resulting maximum *a posteriori* point estimate. As such, the constrained V&S analysis can be viewed as a way to recover the most likely *possible* delay for each individual subject. This may be reasonable for obtaining a low-certainty estimation of the delay for each subject, even though it is biased. However, the analysis becomes highly problematic when these biased point-estimates are amalgamated. The group-level estimate of the mean delay is also biased toward large delay estimates—the posterior for a true delay of zero trials will have some mass greater than zero but none less than zero—but it has several advantages that make the analysis reasonable. The first advantage is that the group-level analysis is explicit about its uncertainty in the form of a distribution and confidence intervals, as opposed to the individual-level delay point-estimates that are treated as certain. The second advantage is that the group-level analysis has substantially greater statistical power, as evidenced by the narrow confidence intervals, implying that the bias is contained within the narrow range of a few trials. This small bias is not as problematic as the much larger bias introduced by fitting delayed exponentials to individual data, which is likely on the order of tens of trials because of the substantially greater noise and drift in the individual data.

### Trial-by-trial decay with random drift explains the observed distribution of delay estimates

While the estimated delay parameters from the individual-level analysis were largely distributed near zero, they were occasionally very large in amplitude. We noticed, however, that this tended to occur in cases where the fit quality was poor. In fact, individuals well-fit by the delayed exponential (R^2^>0.5, corresponding to dots in the top half of Fig 6D) tended to have best-fit delays near zero, whereas individuals less-well fit (dots in the bottom half of Fig 6D) had delay estimates that appear to be distributed somewhat uniformly throughout the experiment window. This suggests that noise dominated the fitting procedure for the less-well fit individuals, making the fitted parameter estimates unreliable. Note that if subjects did indeed have different delays, we would instead expect the maximum R^2^ values to occur when the delays are near the middle of the fitting window, around trial 90, since this is the delayed exponential that maximizes the variance over a constant function.

**Fig 7:**
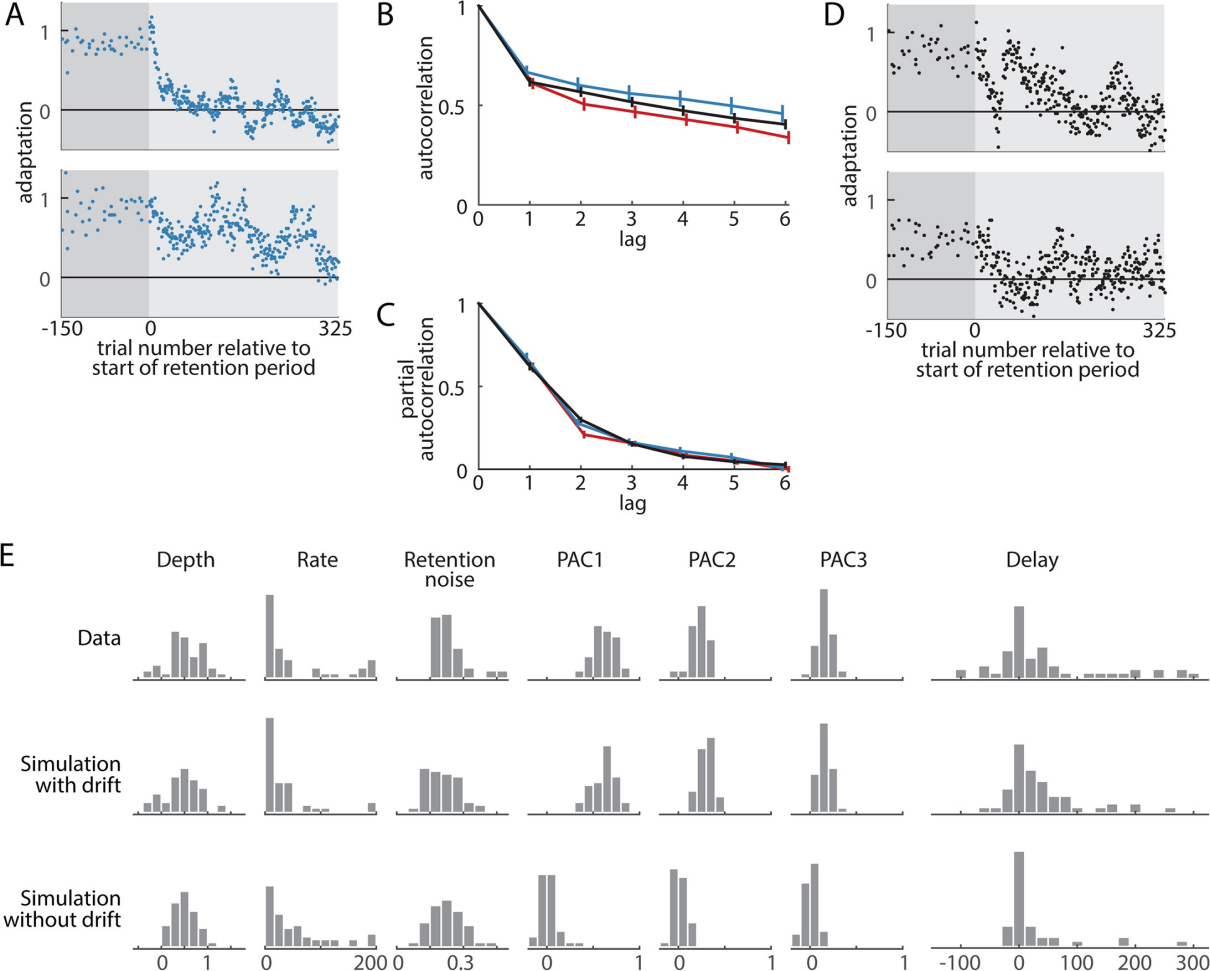
The effect of drift in the retention period on the individual-level delay estimates. We hypothesized that the poor fitting and large delays we found in some subjects in Fig 6D were due to random drifting noise in the retention period. (A) Two example subjects from the zEC shooting movement experiment show pronounced drift (large persistent deviations) in the retention period. (B-C) The autocorrelation function (B) at lag τ represents the raw correlation between trials separated by τ, i.e. between trial t and t−τ. The partial autocorrelation function (C) measures the correlations for trials separated by τ adjusting for the effects of the intermediate trials (see Methods). Independent noise will have autocorrelation and partial autocorrelation functions equal to zero. The red and blue traces from experiments 1 (vEC) and 2 (zEC) have autocorrelations consistently greater than zero, thus showing there is correlated noise (drift) present during the retention period data. The black lines are the result of a simulation designed to match the correlation structure of the data. The simulations included 60 subjects, each decaying with zero delay, and with individual differences in decay depth, decay rate, and noise level (see Methods). We then fit these simulations with delayed exponentials to determine the effect of the drift on the resulting distribution of delay estimates. (D) Two example simulated subjects show realistic drifting behavior, comparable to that in panel A. (E) Both the simulations with and without drift have similar histograms to the experimental data for decay depth, decay rate, and standard deviation of noise during the retention period (retention noise). As expected, the partial autocorrelation function at lags 1–3 (PAC1–PAC3) are different for the drift and no-drift simulations with the drift simulation matching the data. The simulation with drift results in delay estimates that have a distribution similar to the experimental data: largely centered at zero with a wide spread and several subjects with very large delay estimates. In contrast, the simulation without drift has a much narrower distribution of delays and fewer large delay estimates. This shows that the amount of drift present in the data is capable of causing best-fit delays to be very large even when the true delay is zero.

We hypothesized that both the poor fitting and the large estimated delays were caused by random drifting noise in adaptation levels during the retention period, as such drift is readily apparent in many subjects’ data, including all example subjects shown in Figs 6B and 7A. The vEC data in Fig 6 had the average response to the vEC sequence subtracted from the individual data, so the apparent drift is not likely to be driven by vEC sequence specific components, although individual differences in learning rates and stiffness could cause some residual vEC-specific patterns to remain. However, drift was also clearly present in zEC subjects (Fig 7A) who did not have errors during the retention period, so the observed drift could not be merely explained by the presence of an externally-imposed error sequence.

We characterized drift during the retention period using the autocorrelation and partial autocorrelation functions. The autocorrelation function at a given lag τ measures how trials separated by τ are correlated. Thus, if adjacent trials are strongly correlated, then trials two apart will also be. The partial autocorrelation corrects for this by describing how trials separated by τ are correlated after adjusting for correlations expected from all the intermediate trials, thereby producing an estimate of the individual contribution of each lag to the observed correlation structure. The retention periods from experiments 1 and 2 show strongly positive autocorrelation and partial autocorrelation, thus showing there is significant drift in these retention periods since independent noise predicts both these functions to be zero. Furthermore, the autocorrelation and partial autocorrelation functions are consistent between the two experiments (Fig 7B and 7C) with the only significant difference in the partial autocorrelation function occurring at lag-2, where the vEC data is 25% less than zEC. This single difference in the partial autocorrelation at lag-2 gives rise to a fixed gap between the vEC and zEC autocorrelation functions at lags of 2 and above.

To explore how sensitive the individual-level curve fitting procedure for delay estimation might be to the drifts in the individual subject data, we simulated a decay process either with or without drift. To make the simulations realistic, they also included individual differences in decay depth, decay rate, and random (white) noise, with parameters drawn from population distributions derived from our data. We characterized the drift using an autoregressive moving-average model (see Methods). We chose the drift parameters to match the autocorrelation and partial autocorrelation functions of the data (Fig 7B and 7C), thus ensuring the simulations had similar noise correlations to the data. Importantly, all simulations included immediate decay in the retention block. The two example simulated subjects in Fig 7D suggest that this model captures realistic drifting behavior. More rigorously, the autocorrelation and partial autocorrelation functions of the drift model agree well with the data (Fig 7B and 7C).

Using an unconstrained individual-level fitting of the decay onset delay, this zero-delay simulation with drift largely reproduced both the width of the delay estimate histogram derived from the data (interquartile ranges of 61 trials for the data vs 52 trials for the simulation with drift) as well as having several extreme-valued outliers (Fig 7E, right panel). Removing drift from the simulation while retaining individual differences in decay depth, decay rate, and random noise resulted in a much narrower spread (interquartile range of 9 trials) with only a few extreme-valued simulated subjects who were poorly fit due to having small decay magnitude. The simulations with drift also displayed similar population histograms to the experimental data for decay depth, decay rate, retention period noise, and the first few partial autocorrelation function values, suggesting that the data could have arisen from such a model (Fig 7E). This analysis shows that a zero-delay trial-by-trial decay model that includes random drift can account for the pattern of individual participant delay estimates observed in the experimental data.

## Discussion

We examined the context-dependent decay hypothesis for the decay of motor adaptation by expanding and refining the experimental design and data analysis presented in the Vaswani and Shadmehr ([14], V&S) paper that first proposed this hypothesis. These additional experiments and analyses provide a clearer view of decay behavior, but they also eliminate previous support for the context-dependent decay hypothesis.

One line of evidence for the context-dependent decay hypothesis was the lack of decay observed when vEC trials were used to mask the transition from a training period to a retention period [14]. As in the V&S study, we used a vEC-based retention period to effectively match several key features of the training environment, thereby masking the context change to a similar degree (Fig 2). However, we nevertheless found clear evidence for robust decay during vEC-based retention, and we found that the amount of decay was essentially identical regardless of whether the context was masked (vEC-based) or not (zEC-based) (Figs 3 and 4).

The most direct evidence for the context change hypothesis would be the demonstration of an interval of full retention before participants detected the changed context, i.e. a delayed decay onset. Previous findings suggested this delay to already be 40 trials on average in an ordinary zEC retention period, ballooning to over 90 trials in a vEC-based retention period where the context change is masked [14]. However, using a statistically-powerful group analysis, we estimate the delay to be zero trials for both vEC and zEC-based retention, with 95% confidence that the delay is no more than 1 trial in both cases (Fig 5). An individual subject analysis supports this result, estimating the distribution of individual delays to be centered near zero trials for both vEC and zEC-based retention (Fig 6). These findings show decay to be independent of context change detection and instead suggest that continual incremental decay is an inherent component of error-based learning.

### Comparison to the Vaswani and Shadmehr results

Given that the experiments performed here were largely inspired by those that V&S conducted, and in some cases were directly derived from them (the +FF arms of experiments 1a and 2), it may seem somewhat surprising that the findings disagree so strongly. Fortunately, the current results shed light on why this is the case. One key issue is that V&S constrained delay estimate parameter, λ, to be after retention period onset (λ≥0), but we show this causes biased estimates of delay in the presence of noise, especially if true delays are near zero (Fig 6A). Moreover, we find clear evidence of drifting (correlated noise) behavior superimposed on the decay curves in our data and show that these drifts increase the spread of the distribution of delay estimates (Fig 7), which would amplify the delay estimation bias when coupled with the λ≥0 constraint.

Our data also provide insight into why V&S reported a lack of decay for vEC-based retention. We found 3 main issues obscuring the decay in the vEC retention period. The first issue is that all participants in the previous study used the same vEC error sequence, resulting in vEC-based retention curves that were obscured by a large irregular pattern of oscillations due to stiffness- and learning-related motor responses driven by the specific vEC sequence employed. Grossly, these effects manifest as what looks like noise superimposed on the retention curves that makes the true retention curves difficult to discern (see Fig 3A and 3B). Interestingly, these effects were acknowledged but not accounted for in the V&S study. Here we found that balancing the vEC sequence effectively cancelled out the vast majority of the trial-to-trial variance in the mean decay curve, revealing clear decay even in the unreferenced +FF data that showed weak learning. The second issue is that the previous study examined vEC decay only following +FF training, with the result that learning and decay were both underestimated compared to what would be observed if participants were balanced across +FF and −FF training. Our unreferenced +FF data showed weak learning and decay, but our unreferenced −FF data showed strong learning and decay, making it clear that strong decay can occur in a vEC-based retention period that masks context change. Finally, the third issue is that the previous study examined vEC decay without reference to the behavior expected following null FF “training” as a control. We show that subtracting the baseline response to null FF training, measured in experiment 3, reveals strong decay in both FF directions (Fig 4). Thus we show that either (1) balancing the vEC sequence (Fig 3), (2) balancing +FF and −FF training (Fig 3), or (3) control-referencing the decay curve by subtracting the null FF training baseline from it (Fig 4) reveals a robust pattern of learning and decay in both zEC and vEC data.

An additional issue is that V&S masked context change with vEC-based retention only for +FF *shooting movements;* however they primarily compared these results to zEC-based retention for +FF *point-to-point movements*. Our results show that failing to equate movement type in this comparison would inflate the apparent zEC vs vEC difference for unreferenced data because unreferenced +FF point-to-point data display somewhat greater zEC decay than unreferenced +FF shooting data (Fig 3). When we compared vEC-based retention to zEC-based retention within the same movement type, we found essentially identical decay for vEC and zEC trials.

In summary, we suggest that the differences between the current findings and those reported in V&S primarily arise from better-controlled experimental design (balancing the vEC sequence and FF orientation) and analysis (referencing the decay curves to an appropriate baseline, comparing decay within rather than across movement types, and using an unbiased estimate of decay onset delay) in the current study.

### Drifts in motor output during the decay of motor adaption

Like V&S, we found that fitting delayed exponential functions to individual subject data resulted in a few very long best-fit delays. However, these extremal fit parameters were sometimes positive and sometimes negative, and the goodness of fit in these cases tended to be poor, suggesting parameter estimation was unreliable. Interestingly, we also observed a clear pattern of drifting behavior in the individual decay curves (Fig 6B), which would decrease the fidelity of individual parameter estimates and also potentially give the visual impression of delayed decay in a small number of individuals. Further analysis revealed the amount of drifting behavior present in our data to be sufficient to broaden the distribution of estimated delays to agree with the data and to account for the small number of very long best-fit delays even if there were no true delays in decay onset (Fig 7). These drifts largely cancel in the group-averaged data so they have much less effect on the decay magnitude estimates and the group-level analysis of delay.

### Intrinsic trial by trial decay

Further support for the intrinsic trial-by-trial decay hypothesis is provided by the widespread observation that asymptotic motor adaptation is incomplete, generally reaching levels 10–30% short of what would be required for a complete compensatory response [10,17–24]. This results in consistent non-zero asymptotic errors that are not further reduced by additional motor adaptation. One possible explanation for the persistence of these errors is that they are too small to drive additional adaptation. However, small errors have been shown to drive proportionally larger adaptive responses than large errors [25–27]. Furthermore, the size of the asymptotic error is not fixed but scales with the size of the perturbation [28,17]: large perturbations have large asymptotic errors, yet similar errors are compensated for on small perturbations resulting in smaller asymptotic errors. This implies that the motor system is in fact sensitive to those larger errors, yet is unable to compensate for them when the perturbation is large. Since the motor system seems to be attempting to eliminate these asymptotic errors, stable but incomplete asymptotic adaptation can only be achieved as an equilibrium between the new learning on each trial (usually 5–20% of the remaining error [2,19,29]) and the same amount of decay on each trial. This idea is further supported by a recent study from van der Kooij et al. [30], who manipulated the balance between new learning and decay by magnifying or shrinking endpoint feedback during learning, which should affect the learning on each trial but not the decay. They demonstrated that when the error signal was magnified, thereby increasing the drive for error-dependent learning, asymptotic adaptation increased compared to veridical error display because the stronger learning required less true error to balance the decay. They also demonstrated the complementary finding of weaker asymptotic learning for error-signal shrinkage. The authors interpret this as support for the idea that incomplete adaptation arises from an equilibrium between new learning and decay, in line with the intrinsic decay hypothesis.

In contrast, the intrinsically-stable adaptation posited in the context-dependent decay hypothesis would result in perfect retention (*A = 1* in state space models) if no context change were detected, as would be the case during the late training period. With perfect retention, there would be no decay to balance the new learning, so learning would proceed to fully compensate for the perturbation at asymptote, resulting in no asymptotic error, contrary to widespread experimental observations showing incomplete learning and non-zero asymptotic error.

While intrinsic decay may seem like a bug preventing the maintenance of stable memories, it can instead be seen as a useful feature that supports robust motor learning. Transient motor memories are an appropriate response to transient changes to the environment such as donning eyeglasses that will soon be removed, performing limited-duration tasks, or adjusting to changes in motor output ability arising from transient muscle fatigue. Matching the persistence of a motor memory to the persistence of the environment allows ideal flexibility and compensation [31]. A possible mechanism for matching the rate of decay to the durability of the corresponding sensorimotor stimuli is a multi-rate learning model [2].

A second feature of intrinsic decay is that it provides a mechanism for optimizing the tradeoff between energy and task success [32,33]. Task success is prioritized by learning from errors, while energy is minimized by decaying back to a lower-energy state. An equilibrium is formed when the additional task success is no longer more important than the increased energy cost, which occurs when the learning and the decay balance. While this idea is particularly attractive in the case when decay is directed toward a lower-energy state, as is the case in FF learning where decay serves to reduce the lateral force produced, it is less clear how decay toward a same-energy state optimizes energy expenditure. For example, decay in visuomotor rotation tasks is generally directed toward baseline performance of no rotation, despite the fact that there is no additional energetic cost associated with the rotated movement [3,7,8,11]. One explanation is that energetic costs in these cases may be associated with changing the more permanent neural mappings to reflect the new learning.

### Context-dependent decay

Previous support for context-dependent decay hinged on 2 key predictions: delayed decay and increased delays with masked context changes. The group-level decay onset analysis illustrated in Fig 5 establishes that individuals are not slowly accumulating evidence that the context has changed, the signature of context-dependent decay as it has been proposed, because slow accumulation is at odds with the decay onset latencies of 0–1 trials that we found. We also show that decay onset is very similar for the unmasked zEC and masked vEC retention periods (Figs 5 and 6), using the same masking procedure that provided the previous support for the context-dependent decay hypothesis. Therefore, our results contradict both predictions that the context-dependent decay hypothesis previously advanced and leave the hypothesis without support.

Still, it is impossible to prove that the decay is not driven by some change of context. Indeed, an environment that probes the retention of previous training must be different in some way than the environment used for training, so there will always be a change of context between training and retention environments that could conceivably account for differences in behavior observed. Thus the relevant question is whether there is, in fact, a tangible difference in behavior between training and retention periods that is not caused by the removal of the training signal itself (i.e. the error signal that drives continued learning). The current experiments find no evidence for such a difference, but this cannot, of course, preclude future work from demonstrating one. What we have specifically shown here using vEC vs zEC-based retention blocks is that large differences in error variability and reward frequency have little effect on the retention of a learned adaptation, in contrast to what was previously suggested. Thus if context change detection is required for the decay of motor adaptation, the mechanism for this detection must be both exceedingly rapid (resulting in a latency ≤ 1 trial) and based on contextual information that is not yet understood.

## Methods

### Ethics statement

All experiments were approved by the Harvard University Institutional Review Board. All subjects provided informed consent to participate.

### Participants

One hundred and thirty subjects (mean age 22.1±5.2, 71 female) with no known neurological impairment participated in the study. One hundred and twenty eight were right handed, and all subjects used their right hand for the experiments.

### Experimental paradigm

Subjects performed 10cm reaching movements to a 1cm diameter target while grasping the handle of a 2-link planar robotic manipulandum, which produced forces and measured kinematics at a rate of 200Hz. The targets and veridical visual feedback of hand position, in the form of a 3mm-diameter cursor, were displayed on a computer monitor with a refresh rate of 75Hz.

Experiments 1–3 were based on shooting movements that continued past the target without stopping (Fig 1). The movement was brought to rest by a “virtual pillow” that began 1cm beyond the target. This pillow was implemented as a resistive viscous force field with a viscosity increasing with distance beyond the target at a rate of 30N/(m/s) per additional centimeter beyond the target. 300ms after coming to a complete stop, the robotic manipulandum automatically moved subjects back to the starting point for the next movement. Movements that passed within 6mm of the target center between 150ms and 250ms after movement onset, which was defined by a velocity threshold of 5cm/s, were rewarded by a pleasant sounding chirp and brief animation of the target exploding on the screen. Movements passing within 6mm of the target center, but outside of the designated times, were labeled too fast or too slow by instead recoloring the target red or blue, respectively, without a sound. No visual or auditory feedback was given on trials that failed to pass within 6mm of the target center.

Experiments 4–6 were based on point-to-point movements, which stopped at the target. These movements are described in the supplementary materials (S1 File).

During each movement, the robotic manipulandum applied forces in one of three ways. During null field trials, no forces were applied. During force field (FF) trials, a force proportional and orthogonal to the movement velocity was applied: for a given movement velocity $\vec{\dot{x}}={\left[\dot{x}\;\dot{y}\right]}^{T}$, the robot produced a force $\vec{F}={\left[F_{x}\;F_{y}\right]}^{T}$ equal to $\vec{F}=B\vec{\dot{x}}$, where $B=\left[\begin{array}{cc} 0 & b\\ -b & 0 \end{array}\right]$ defines a curl force field. The sign of the scalar parameter *b* dictates the direction of the force field. The +FF subgroups experienced FFs with *b* = +15N/(m/s), whereas the −FF subgroups experienced FFs with *b* = –15 N/(m/s). The third way the manipulandum applied forces was through error clamp (EC) trials [1,2,6,10,12,14,34,35]. During EC trials, reactive forces effectively constrained motion to a straight-line path. These reactive forces acted to create a virtual spring with stiffness of 6000N/m and damping of 250N/(m/s) in the axis orthogonal to the path constraint (Fig 1).

### Variable error clamp movements

Zero-error clamp (zEC) trials constrained the movement to go straight to the target, clamping the lateral error to zero. Consequently, transitioning from FF training to a zEC retention block results in sharply decreased movement direction variability and increased reward frequency.

To mask the context change from training to retention blocks, we used a retention block composed of variable error clamp (vEC) trials. While zEC and vEC trials both constrained the movement to a specified straight-line path, the vEC path direction varied subtly from one trial to the next. The direction for each trial was drawn from a zero-mean Gaussian distribution with a standard deviation of 2.6°, similar to the variability observed late in FF training and matching the variability used by V&S.

We used both vEC and zEC trials to measure the retention of adaptation throughout the shooting movement retention period. However, vEC trials suffer from a stiffness-related bias introduced by their directional errors: subjects are trying to move straight forward but are forced to move at a non-zero angle, resulting in extra force toward the middle that scales with the subject’s stiffness. To estimate this bias, we used a linear model to estimate the relationship between the error clamp direction and the residual errors, computed as the difference between the adaptation coefficient and the smoothed adaptation using only zEC trials (moving average filter, 25 trial window). The result was a −0.03 change of adaptation coefficient per degree of angular error.

However, having removed the stiffness-related bias, large swings in adaptation remained during the vEC period of a pilot study. The swings arose when the vEC error sequence included several large errors consecutively in the same direction, indicating that subjects were learning from the errors. To minimize these effects, we selected an error sequence by randomly generating candidate sequences, then rejecting sequences that had consecutive patterns of large errors, quantified using the sequence’s binary error pattern (−1 if the direction misses the target to the left, +1 if it misses the target to the right, and 0 if it hits the target). Specifically, a sequence was rejected if the 5-trial smoothed binary error pattern exceeded a magnitude of 0.5, the 9-trial smoothed pattern exceeded a magnitude of 0.2, or the maximum gap between consecutive rightward or leftward misses exceeded 25 trials. This algorithm rejects all but about 1 in 100 thousand randomly generated sequences.

Having generated a sequence satisfying the above constraints, we used that sequence (experiment 1a) or its mirror opposite (experiment 1b) for all vEC experiments. This design allowed us to infer the extent to which vEC-driven learning contaminates the individual subject decay curves by comparing experiments 1a and 1b as well as remove these effects by averaging subgroups 1a and 1b together. This balancing of the vEC direction removes the effects of stiffness without the need for an explicit model, so we did not separately remove the stiffness-related bias from the data presented here.

The vEC-sequence specific effects will result in noisier, less smooth decay curves. To quantify the smoothness of the decay curves, we used trial-to-trial variance, computed as the variance of the trial-to-trial changes in adaptation. Equivalently, if *d*(*t*) is the running difference between the adaptation at trial *t* and at trial *t−1*, then the trial-to-trial variance is the variance of *d*.

### Adaptation measures

For the first part of the analysis, the force produced during an error clamp was quantified using the adaptation coefficient measure, which regresses the force produced onto the ideal compensatory force in a positive-FF environment. For positive subgroups, a perfect force production would yield an adaptation coefficient of 1; for negative subgroups, a perfection compensation would yield an adaptation coefficient of −1. This measure has been used extensively [2,6,10,12,14,17,34,35], particularly with point-to-point movements.

For the second part of the analysis, the force produced during an error clamp was quantified using the integrated lateral force measure, which integrates the lateral component of the compensatory force pattern and normalizes it by the integrated ideal force pattern for a positive FF. As with the adaptation coefficient measure, ideal performance yields an integrated lateral force measure of +1 for +FF trials and −1 for −FF trials. Unlike the adaptation coefficient measure, this measure is not sensitive to the specific timing of the compensatory force pattern.

### Experimental procedures

Experiments 1–3 used shooting-movements in the 90° direction (away from the body) (Fig 1D). Experiment 1 (n = 40; 2x2 design with force field direction (+FF vs. −FF) x vEC sequence (1a vs. its mirror opposite 1b); 10 subjects did each of the 4 permutations) consisted of a training block composed of 300 FF trials followed by a retention block of 325 vEC trials. Experiment 2 (n = 20; 10 positive FF, 10 negative FF) was identical, except that the retention block used zEC trials. Experiment 3 (n = 10) served as a control experiment, with a 300-trial null field (0-FF) training block followed by a 300-trial zEC retention block.

In each of experiments 1–3, a randomly-selected 20% of the trials in each block, including the vEC retention blocks, were replaced by zEC trials to allow adaptation to be accurately measured throughout the experiment.

We balanced FF direction (positive versus negative) in all applicable experiments (experiments 1 & 2). This was not an issue in the control experiment (experiment 3) where “training” was performed using a null FF. Additionally, we balanced experiment 1 against the vEC sequence of errors by using the mirrored error sequence for half the subjects (experiment 1a vs 1b).

Experiments 4–6 were analogous to experiments 1–3 using point-to-point movements. See supplementary materials for details (S1 File).

### Quantifying decay

The decay of motor adaptation refers to the loss of previously-learned adaptation during a retention period in which the trained perturbation (FF) is withheld. Its overall magnitude can be quantified as the difference between the adaptation levels observed in late training and late retention.

By balancing the experiment across FF directions, the fractional decay for the entire experiment can be estimated by comparing the difference between subgroups during the late learning and the difference during late retention. Specifically, the overall decay for an experiment is $\frac{\left(LP-LN)-(RP-RN\right)}{LP-LN}$, where LP and LN refer to the asymptotic learning (mean of last 150 trials in experiments 1, 2, and 4; mean of last 100 trials in experiment 5) in the positive and negative subgroups, respectively, and RP and RN refer to the asymptotic retention (mean of all the retention trials after the first 150) in the positive and negative subgroups, respectively. Error bars for this overall decay estimate were calculated using a bootstrap procedure. Calculating the decay in this way accounts for both baseline and learning asymmetries between the FF directions.

### Individual participant analysis of decay onset latency

The context-dependent decay hypothesis predicts that the learned adaptation will decay suddenly as soon as the context change is observed, but not before. Considering a window around the late training block and the entire retention block, the predicted shape of the adaptation curve would then be a delayed exponential function,

$$f(t)=\{\begin{array}{r} a,\;\text{if}\;t\leq \lambda \\ (a-b)\text{exp}\left(-\frac{\left(t-\lambda \right)}{\tau }\right)+b,\;\text{if}\;t>\lambda \end{array}$$ (Equation 1)

We fit the individual subject data using this function using some mild constraints in order to capture the proposed behavior. The constraints were,

$$\begin{array}{c} \left|a\right|<1.5\\ \left|b\right|<1\\ 2\leq \tau \leq 200 \end{array}$$

The constraint on τ was derived from the literature, where decay rates have previously been estimated in the range of 100–150 trials [1,2]. V&S used the same constraint on τ but were more restrictive of the asymptotic learning and decay levels, with *a* constrained between 0.5 and 1.5 and *b* between −0.3 and 0.3.

Leaving the delay parameter λ unconstrained is particularly important. In order to show the context-detection hypothesis is true, there must be evidence against the null hypothesis that the decay occurs continuously and therefore starts immediately. If all subjects do decay immediately but with noisy motor output, the unconstrained fitted values of λ will be dispersed around the true value of λ = 0 trials after the training block. If λ is observed to be biased toward positive values, then there is evidence for decay occurring systematically later in the retention block, supporting the context-dependent hypothesis. This evidence would be completely obscured if the constraint λ≥0 was used, as in V&S, since even the null hypothesis would be biased toward positive delays. To illustrate this point clearly, we did a simple simulation of 10,000 subjects who had a delay of zero trials, each with asymptotic learning of 0.85, asymptotic retention of 0.40, decay rate τ = 40, and independent noise with standard deviation 0.2; these values represent the mean unconstrained fit parameters in our experimental data. We then fit these simulated subjects using an approach that constrained the delay parameter to be positive and compared the results to an unconstrained fit.

The same-trial stiffness-related deviations and next-trial motor adaptation-related deviations introduced by vEC directional errors (see Methods: Variable error clamp movements) corrupt the quality of fit used to estimate the delay parameter and, correspondingly, the estimates themselves. To mitigate this effect, we removed the average vEC-driven deviations in performance from each subject. The deviations were estimated as half the difference between experiment 1a and 1b mean retention curves, which used mirror-opposite vEC sequences.

We used nonparametric statistics throughout the analysis of individual-level fits because the resulting delay distribution included particularly heavy tails. We were therefore not able to justify the assumption of a Normal distribution necessary for the parametric tests we used in our other analyses.

### Estimating decay onset at the group level

Here we leveraged the statistical power of group data to better estimate the mean individual delay using all subjects pooled from the +FF and −FF subgroups, after flipping the −FF data. As with the individual-level approach, we first removed the average vEC-driven performance deviations from each subject (see section above).

We could not simply examine the mean data to determine the average decay onset delay since the mean data would begin to decay early even if only a fraction of the population decayed early, as would be expected if the distribution of delays was exponential as posited by V&S. We therefore split the data into two subgroups, those who decayed most vs least in the first 50 trials of the retention period relative to their asymptotic learning levels, in order to separate the short delay from the long delay individuals. The idea is that the low-decay subgroup should be predominantly composed of long-delay subjects if there were any. We chose the length of the dividing window (50 trials) as a balance between wanting a long window to reduce noise and wanting a short window for more sensitivity to small mean delays, as very long windows begin to partition subjects based on decay depth rather than decay onset time. The 50 trial window was chosen since it is a reasonably long window but should still select based primarily on decay onset time rather than decay depth since it is less than the median value of 63 trials that would result from an exponential distribution of delays with a mean of 90 trials, as reported in V&S for a vEC-based retention period. Thus, if delays were distributed as reported in V&S, the low-decay subgroup would be largely composed participants with delays greater than 50 trials, resulting in essentially no decay during the 50-trial decision window and decay onset sometime thereafter.

The ratio of early decay to late decay, which we refer to as the early decay ratio, would decrease as decay onset latency increases. We thus used this early decay ratio as a measure for characterizing decay onset latency:

$$\text{Early Decay Ratio}=\frac{\text{Early Decay}}{\text{Late Decay}}=\frac{\text{Learning}-\text{Early Retention}}{\text{Learning}-\text{Late Retention}}.$$

Here early retention is taken to be the mean adaptation measured over the first 50 retention period trials, late retention is measured over the last 75 trials of the retention period, and learning is the asymptotic learning level in the last 50 trials of training. A low early decay ratio is indicative of delayed decay. The early decay ratio was computed for both the high and the low (median-divided) decay subgroups.

We ran simulations to provide references for the early decay ratios derived from the data. We simulated subjects using population parameters for a delayed exponential decay (Eq 1): a~N(0.875,0.100^2^), b~N(0.360,0.200^2^), and τ~N(40,10^2^), with independent noise distributed N(0,0.25^2^) on each trial. The distributions of a and b match the late training (last 150 trials) and late retention (last 175 trials) levels in the shooting movement data, while the decay rate τ and noise were based on the unconstrained fits to the shooting movement data, although the noise is an overestimate of the noise in our data in order to broaden the likelihood functions, thus providing less evidence relative to the prior and making this analysis more conservative. We also used an exponential distribution of delay parameters, λ~Expo(μ_λ_), with μ_λ_ ranging from 0 to 90 trials for the various simulations. We ran 1000 simulations for each mean delay μ_λ_, where each simulation used the same number of subjects as the corresponding experiment (40 when the simulations provided a reference for experiment 1, 20 for experiment 2). For a given mean delay μ_λ_, each of the 1000 simulations provides an estimate of the early decay ratio for both the high and low decay subgroups. Thus, the 1000 simulations at a given mean delay provide an empirical joint probability distribution over the early decay ratio for the high- and low-decay subgroups, and as such, serve as a likelihood function for the mean delay parameter: P(ED_high_, ED_low_| μ_λ_) for the early decay ratios ED_high_ and ED_low_ for the high and low decay subgroups.

We used a discrete uniform prior distribution over the trials 0 to 90 in order to estimate confidence intervals for the mean individual delay parameter, μ_λ_. The posterior distribution over the mean delay was therefore *P*(*μ*_*λ*_ | *ED*_*high*_, *ED*_*low*_) ∝ *P*(*ED*_*high*_, *ED*_*low*_ | *μ*_*λ*_)*P*(*μ*_*λ*_).

### Characterizing the drift in the data

The autocorrelation function is the correlation between a time series and the same series shifted by a given lag: corr(*x*(*t*), *x*(*t* – *τ*)) for lag τ. The partial autocorrelation function, a more specific measure, is the additional correlation between *x*(*t*) and *x*(*t* − *τ*) after taking into account the lag-τ correlation that would be expected given that *x*(*t* − *τ*) influences the intermediate values *x*(*t* − 1), *x*(*t* − 2), …, *x*(*t—*(*τ* − 1)) through lower-order autocorrelations, which themselves influence *x*(*t*) through lower-order correlations. Put another way, the lag-τ partial autocorrelation is the parameter estimate for *x*(*t* − *τ*) when regressing *x*(*t*) onto all elements of the set {*x*(*t* − 1), *x*(*t* − 2), …, *x*(*t*− *τ*)} simultaneously. It therefore represents the correlation between *x*(*t*) and *x*(*t* − *τ*) adjusting for *x*(*t* − 1) through *x*(*t−*(*τ−1*)).

In order to model the drift in the retention period, we fit a model with one autoregressive parameter and one moving average parameter, which takes the form,

x(t)=AR·x(t−1)+ε(t)+MA·ε(t−1)

for an autoregressive coefficient AR, moving average coefficient MA, and independent white noise process ε. This model was fit to the individual subject data, from retention trial 76 onward, after subtracting out the across-subject mean retention at each trial, which was done to focus the autoregressive models on the drift behavior instead of the mean decay behavior. That said, similar results were achieved if the entire decay block was considered.

### Generative model of trial-by-trial decay with random drift

We used a generative model of trial-by-trial decay to understand how drift and noise affect the individual fitting procedure. The model included six parameters for each simulated subject: amount of decay D~N(0.56,0.28^2^), decay rate τ = 35.8, late learning trial-by-trial white noise with standard deviation σ = 0.14, and an autoregressive moving average model with AR~N(0.92,0.04^2^), MA~N(−0.52,0.05^2^), and stdev(ε)~N(0.17,0.04^2^) to induce random drift behavior. Each simulated subject, *y*_*sim*_, was generated using the following dynamics:

$$\begin{array}{l} \;y(t)=\{\begin{array}{r} D,\;\text{if}\;t\leq 0\\ D\cdot \exp(-t/\tau ),\;\text{if}\;t>0 \end{array}\\ \;n(t)=AR\cdot n(t-1)+\varepsilon (t)+MA\cdot \varepsilon (t-1)\\ y_{sim}(t)=y(t)+n(t) \end{array}$$

with the noise *n*(*t*) during the training period instead replaced by independent white noise, *n*(*t*)~N(0,σ^2^). Critically, the simulations all included immediate decay, corresponding to the null hypothesis of trial-by-trial decay.

We initially chose the decay parameter values to match the values obtained from fitting the experimental data with zero-delay exponentials and the drift model parameters by fitting the drift model to the individual data. However, the simulated autocorrelation and partial autocorrelation functions were far more varied than those of the data, with slightly smaller overall values. This is not particularly surprising because noise in the simulation corrupts the ability to estimate model parameters, thus increasing the spread of the estimated parameters. Thus, to better match the autocorrelation and partial autocorrelation functions of the data, we adjusted the AR term up by 0.04 to 0.93 and the MA term down by 0.05 to −0.55 and reduced the across-subject variance of those parameters by factors of 5 and 10, respectively. Similarly, we treated the decay rate and noise during the training period as the same across subjects since the fitting procedure produced enough variance to match the empirical values without subject differences. Note that narrowing these distributions has the effect of narrowing the best-fit delay distribution so it is not the reason for any large best-fit delays.

We simulated 60 subjects, matching the number of shooting movement subjects in experiments 1 and 2, and fit them with delayed exponentials. We then compared the simulated subjects to the data from experiments 1 and 2 in terms of the distribution of fit delays as well as the distributions of 6 parameters that describe the shape of the decay and the noise structure, as recovered from the simulated and experimental data. We did this both for the simulation described above and an analogous no-drift simulation in which the retention period noise was treated as independent with standard deviation distributed N(0.23,0.07^2^) to match the data.

## Supporting Information

S1 File Point to point movements: Description and experimental paradigm. (PDF)

S2 File Variably directed error clamps reduce the salience of context changes in a point-to-point movement retention block. (PDF)

S3 File Point-to-point learning and decay appear asymmetric across FF directions but are unaffected by context change salience. (PDF)

S4 File Control referencing point-to-point movements reveals symmetric learning and decay but no effect of context change salience. (PDF)

S5 File Control-referenced adaptation quantified using the adaptation coefficient measure for both shooting and point-to-point movements. (PDF)

S6 File Individual-level analysis of point-to-point movements indicates essentially no systematic delay in decay onset. (PDF)
