# Supplementary material for: The Decay of Motor Memories Is Independent of Context Change Detection
Source: PLoS Comput Biol. 2015 Jun 25;11(6):e1004278. doi: 10.1371/journal.pcbi.1004278 (PMC4482542; doi:10.1371/journal.pcbi.1004278)
Supplement: S1 File — (PDF) [file pcbi.1004278.s001.pdf]

S1: Point to point movements: description and experimental paradigm

Experiments 4-6 were based on point-to-point movements that stopped at the target (Fig S1). A movement was rewarded for stopping (tangential velocity <3cm/s) within 6mm of the target center between 400ms and 600ms after movement onset (defined based on a 5cm/s velocity threshold), provided it stayed within 6mm of the target center and below a velocity threshold of 3cm/s for an additional 300ms consecutively. Movement times were identified as correct, too fast, or too slow using the same feedback scheme as for shooting movements.

We implemented the vEC trials for point-to-point movements slightly differently than for shooting movements. Shooting movements are designed to be so rapid that they generally include little or no online feedback correction, whereas slower point-to-point movements can include strong feedback corrections when warranted because their movement time exceeds the sensorimotor loop delay. Correspondingly, the mean movement time for shooting movements was only 216±13ms, while point-to-point movements took 623±59ms in our data, although this total movement time is inflated for the point-to-point movements as it includes corrections to the target. The time when subjects were 80% of the way to the target was 187±13ms for shooting movements and 276±53ms for point-to-point movements, showing that point-to-point movements were much slower and that the point-to-point movements included substantial feedback corrections to arrive at the target, as evidenced by the long time between passing 80% of the way to the target to arriving at the target. A strict EC would counter this feedback correction, resulting in an obvious and immediately detected change of context in the point-to-point movements that is contrary to the purpose of this manipulation. To maintain the ability for subjects to effect feedback corrections in point-to-point movements, we terminated the error clamp at 70% of the target distance and ramped down the error clamp stiffness to zero over the next 100ms from that point to prevent a sudden discontinuity in the applied force.

Experiments 4-6 used point-to-point movements. Experiment 4 (n=10; 5 positive FF, 5 negative FF) used both the 90° and 270° movements for each subject. The experiment consisted of a 300-trial FF training block followed by a 325-trial vEC retention block (Fig S1C). Experiment 5 (n=10; 6 positive FF, 4 negative FF) consisted of movements performed in the 270° direction (toward the body), with return movements occurring in a null field. It was composed of 200 null-field movements, a training block of 160 FF movements, and a retention block of 410 zEC movements (Fig S1C). Experiment 6 (n=40) served as a control and, like experiment 4, was performed in both movement directions. It had a 100-trial 0-FF training block followed by a 100-trial zEC retention block (Fig S1C).

In experiments 4 & 5, a randomly-selected 20% of the trials in each block, including the vEC retention blocks, were replaced by zEC trials to allow adaptation to be accurately measured throughout the experiment. In experiment 6, only two EC trials were included in the training block, occurring at trials 40 and 85. The average force for these two ECs combined across all 40 subjects was used as a baseline for experiments 4 and 5.

We balanced FF direction (positive versus negative) in all applicable experiments (experiments 4 & 5). This was not an issue in the control experiment (experiments 6) where "training" was performed using a null FF.

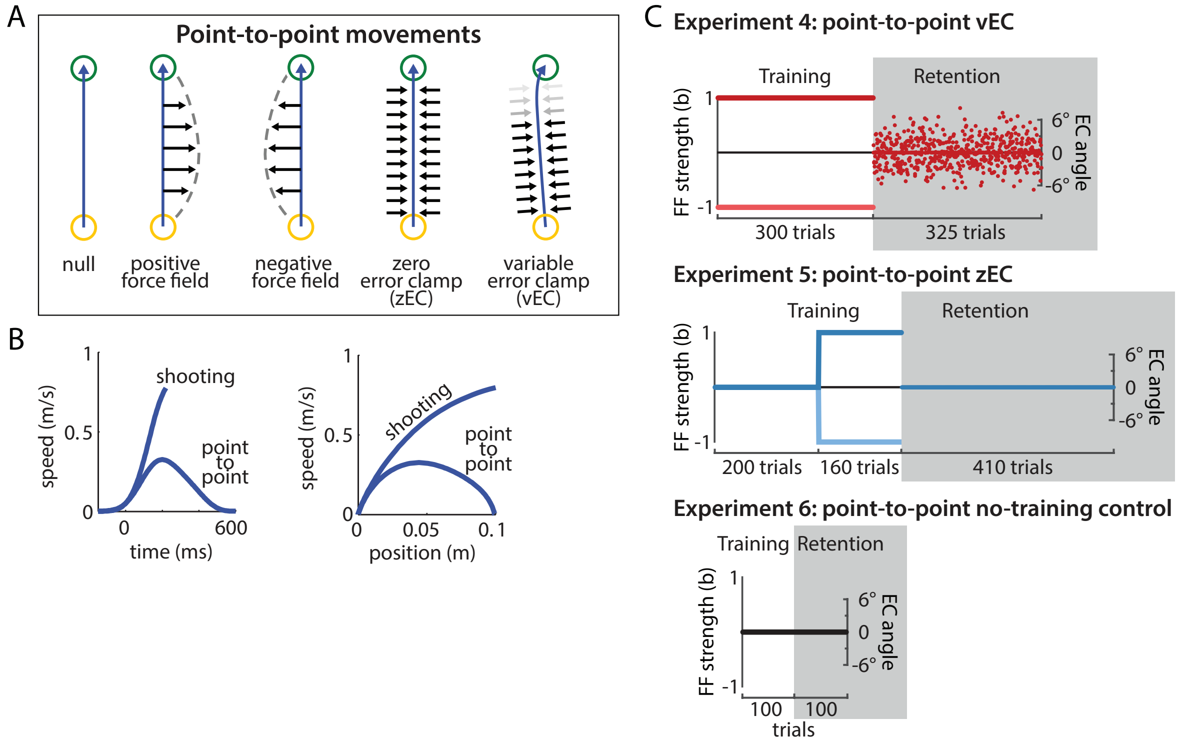

Figure S1: Experimental paradigm for point-to-point movements. (A) Point-to-point movements were to be aimed at the target but brought to rest at the target without robotic assistance. Experiments had a training block consisting of positive force field (+FF) trials, negative FF (–FF) trials, or null trials (0-FF) in which the robotic manipulandum applied forces (black horizontal arrows) proportional to the movement velocity and directed orthogonally to the movement direction. This training block was succeeded by a retention block of error clamp trials, where forces were applied reactively with a virtual channel in order to effectively constrain motion to a predefined straight-line path. Zero-error clamp (zEC) retention trials were always directed toward the target's center, resulting in very low directional variability. In contrast, variable error clamp (vEC) trials were directed along a different non-zero angle on each trial and were used to impose subtle directional variations (σ=2.6°) from one trial to the next during the retention period. The amount of directional variability was matched to the statistics of late FF training, thereby reducing the context change from the training environment. Note that the virtual channel used in point-to-point vEC trials faded over the next 100ms once the movement reached 70% of the way to the target in order to preserve the corrective responses typically observed at the end of point-to-point movements. **(B)** Velocity profiles for shooting and point-to-point movements. Point-to-point movements display bell-shaped velocity profiles, whereas shooting movements reach maximum velocity near the target with profiles more like half bells. The differences in these velocity profiles account for the different force profiles illustrated for the FF movements in Figure 1C vs Figure S1A. (C**) E**xperiments 4-6 were point-to-point movement analogs to the shooting movement experiments 1-3. Each experiment began with a training period of FF trials. For experiments 4 and 5, there were two subgroups (dark and light colors), one training on +FF and the other training on –FF trials; both groups had the same retention periods. Experiment 4 had a vEC-based retention period in which all subjects had the same pre-selected sequence of errors. Note that the force field strength (b) was controlled during the training blocks while the error clamp angle was controlled during the retention blocks. Experiments 5 and 6 had retention periods based on zEC trials. Experiments 4 and 6 were performed in both the 90° and 270° directions, while experiment 5 was only performed in the 270° direction.
