# Supplementary material for: The Decay of Motor Memories Is Independent of Context Change Detection
Source: PLoS Comput Biol. 2015 Jun 25;11(6):e1004278. doi: 10.1371/journal.pcbi.1004278 (PMC4482542; doi:10.1371/journal.pcbi.1004278)
Supplement: S2 File — (PDF) [file pcbi.1004278.s002.pdf]

S2: Variably directed error clamps reduce the salience of context changes in a point-to-point movement retention block

Experiments 1 and 2 were repeated using the more widely-studied point-to-point movement paradigm. Experiment 4 used vEC movements during the retention block, analogous to experiment 1, and experiment 5 used zEC movements, analogous to experiment 2 (Fig S1). The vEC manipulation was instituted similarly, with angles independently drawn from a Gaussian distribution with a mean of 0º and a standard deviation of 2.6º (Fig S1). Compared to the zEC trials of experiment 5, the vEC trials of experiment 4 better matched the directional variability, reward frequency, and trajectory curvature of the late training trials (Fig S2) in a manner similar to the shooting movements. As in the shooting movements, there was no systematic change from training to EC trials in intermovement consistency, measuring the similarity of subsequent movements [14], nor in movement duration for the vEC experiment. However, unlike the shooting movements, the point-to-point movement duration did change between late training and zEC trials because the corrective movements were eliminated. Overall, the vEC-based retention period behaved similarly for point-to-point movements as it did for shooting movements, substantially reducing performance differences between the training and retention periods in our data.

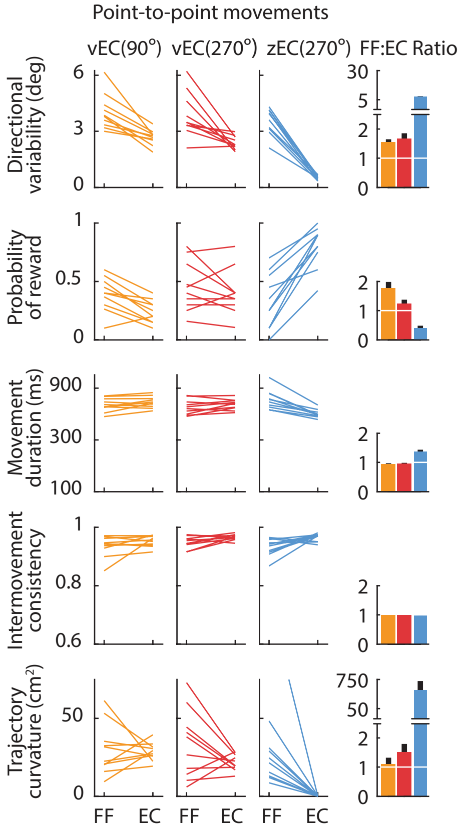

Figure S2: Comparison of point-to-point movement characteristics during late training and early retention trials. Lines connect the average values for the last 20 training trials and the first 20 retention trials for each subject in the point-to-point movement experiments for the 5 movement characteristics that Vaswani and Shadmehr ([13], V&S) used: Directional Variability (Endpoint Standard Deviation in V&S) is the standard deviation of movement angle; Probability of Reward is the observed reward frequency; Movement Duration is the time to the target; Intermovement Consistency measures the similarity of consecutive movements [14]; Trajectory Curvature (Trajectory Deviation in V&S) measures the curvature of the movement, and is the sum of squared lateral deviations from the straight path joining the start and end positions of that path. Subjects could use large differences in these characteristics between the training and retention blocks, as quantified by the ratio of the last 20 training trials to the first 20 retention trials (right-most column), to detect changes in context between these blocks. As with the shooting movements in Figure 2, the point-to-point vEC retention blocks better match the statistics of the training environment than their zEC analogs for all five statistic, suggesting that the context change should be harder to detect. The values and ratios we observe are very similar to those in reported V&S. Error bars show SEM.
