# Supplementary material for: The Decay of Motor Memories Is Independent of Context Change Detection
Source: PLoS Comput Biol. 2015 Jun 25;11(6):e1004278. doi: 10.1371/journal.pcbi.1004278 (PMC4482542; doi:10.1371/journal.pcbi.1004278)
Supplement: S3 File — (PDF) [file pcbi.1004278.s003.pdf]

S3: Point-to-point learning and decay appear asymmetric across FF directions but are unaffected by context change salience

As with the shooting movements, the vEC and zEC data appear similar in both learning and decay. A 3-way analysis of variance (retention period type × FF direction × movement type) reveals that the amount decay is not affected by the presence of vEC vs zEC-based retention periods ($F_{(1,76)}$=0.13, p=0.72), despite clear effects of positive versus negative FF directions ($F_{(1,76)}$=109, p<10^{-15}) and shooting vs point-to-point movements ($F_{(1,76)}$=9.32, p=0.003). Post hoc testing revealed significant decay in all the experimental subgroups (all 8 combinations of vEC vs zEC retention, +FF vs –FF training, and shooting vs point-to-point movements; p<0.02 in all cases). This indicates that vEC-based context change masking failed to prevent the decay of motor adaptation in our data.

Whereas shooting movements exhibited markedly asymmetric learning and correspondingly asymmetric decay across FF directions (Fig 3), analogous point-to-point movement data from experiments 4 and 5 exhibited nearly symmetric learning but asymmetric decay (Fig S3). The vEC-based experiment 4 displayed nearly symmetric adaptation levels (0.74±0.01 vs 0.70±0.03 for the +FF and –FF subgroups in the 270° direction and 0.78±0.02 vs 0.89±0.02 for +FF and –FF subgroups in the 90° direction), as did the zEC-based experiment 5, which was only performed in the 270° direction (0.77±0.04 vs 0.77±0.04 for +FF and –FF, respectively). However, the decay was noticeably asymmetric between the +FF and –FF subgroups in the 270° direction, with experiment 4 data displaying decay levels of 0.39±0.06 and 0.97±0.06 for the +FF and –FF subgroups , and experiment 5 data displaying similar decay levels of 0.29±0.08 and 0.75±0.05, again underscoring the importance of examining decay in a balanced design with both +FF and –FF training that avoids selective sampling of these conditions.

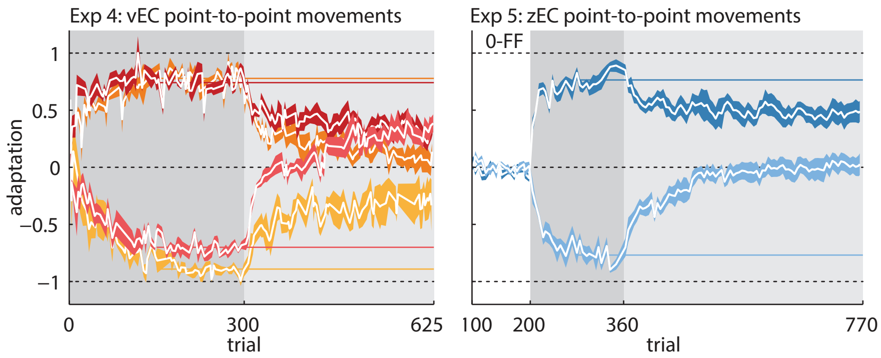

Figure S3: Raw learning and decay in point-to-point movements. As with the shooting movements in Figure 3, the learning and decay curves for the vEC-based experiment 4 data (90° in orange, 270° in red) are similar to the zEC experiment 5 data (270° in blue). Compared to the zEC group, the vEC experiments do not show attenuated decay amplitude, suggesting little effect of context change salience on decay. In both cases, the 270° movements (red and blue) displayed asymmetric decay despite nearly symmetric learning for all groups.
