# Supplementary material for: The Decay of Motor Memories Is Independent of Context Change Detection
Source: PLoS Comput Biol. 2015 Jun 25;11(6):e1004278. doi: 10.1371/journal.pcbi.1004278 (PMC4482542; doi:10.1371/journal.pcbi.1004278)
Supplement: S4 File — (PDF) [file pcbi.1004278.s004.pdf]

S4

Control referencing point-to-point movements reveals symmetric learning and decay but no effect of context change salience

We performed a second control experiment (experiment 6; Fig S1), analogous to experiment 3 but with point-to-point movements, to provide references for the learning and retention curves for the point-to-point data in experiments 4 and 5. Interestingly, control-referencing this data had little effect on the learning and retention curves in the cases where the raw data were fairly symmetric (all of the learning periods as well as the retention period for the 90° vEC data (orange)). However, control-referencing substantially improved symmetry in the conditions with marked asymmetries between the raw +FF and −FF data (the 270° retention periods for the zEC and vEC data (red & blue)) (Fig S4B).

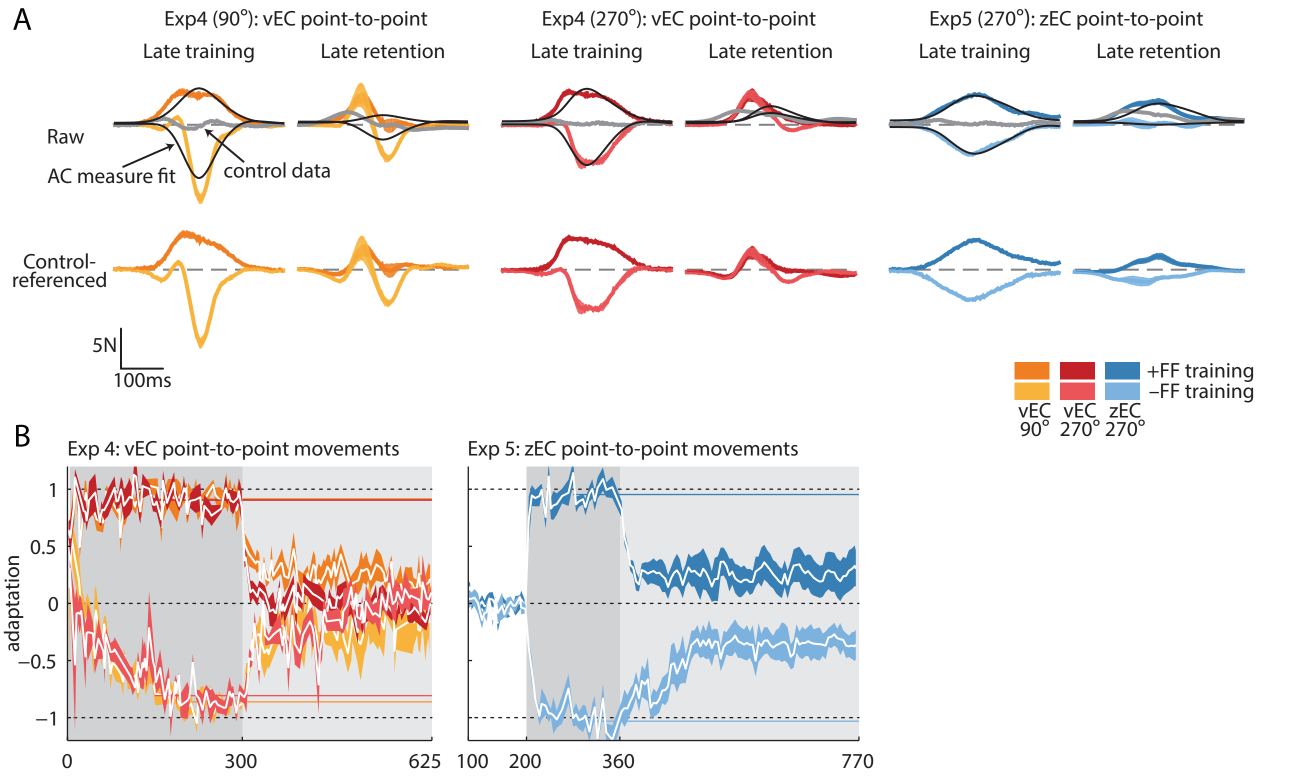

Figure S4: Point-to-point learning and decay referenced to control data from a zero-FF training episode. (A) Top row: raw lateral force profiles for the +FF and −FF subgroups (darker and lighter colors, respectively). Unlike the shooting movement force profiles in Figure 4, the point-to-point force profiles are generally well-captured by the adaptation coefficient measure (black lines) during training. However, the late retention force profiles show a consistent bias toward positive force, especially in the 270° direction. We performed a control experiment consisting of a 0-FF "training" block and a zEC retention block (experiment 6) to provide a baseline reference for adaptation and decay. Considering the force profiles (colored traces in top row) relative to this baseline reference (gray traces in top row) reveals more symmetric adaptation and decay between +FF and −FF conditions for both training and retention (colored traces in second row), as was found in the shooting movements in Figure 4. This occurs because the baseline forces from experiment 6 are small except during the 270° retention period, which is the only condition in which the forces were asymmetric. Yet, the control-referenced retention data is not always well explained by the shape of the adaptation coefficient measure, so we quantified adaptation using an integrated lateral force measure that is agnostic to the shape of the force profile. **(B)** Like Figure S3, the control-referenced vEC and zEC point-to-point data appear similar when quantified using integrated lateral forces (red vs blue). However, here we also see symmetric learning and decay across +FF and −FF conditions in both experiments. As in the shooting movements in Figure 4, the strong decay apparent in both the +FF and −FF arms of the vEC experiment is in contrast to reports of the vEC manipulation eliminating decay.
