# Supplementary material for: The Decay of Motor Memories Is Independent of Context Change Detection
Source: PLoS Comput Biol. 2015 Jun 25;11(6):e1004278. doi: 10.1371/journal.pcbi.1004278 (PMC4482542; doi:10.1371/journal.pcbi.1004278)
Supplement: S5 File — (PDF) [file pcbi.1004278.s005.pdf]

S5: Control-referenced adaptation quantified using the adaptation coefficient measure for both shooting and point-to-point movements

Control-referenced learning and decay quantified by the adaptation coefficient measure is similar to control-referenced learning and decay quantified using the integrated lateral force measure, as it was in Figure 4 (shooting movements) and Figure S4 (point-to-point movements). Learning and decay remain similar between analogous vEC and zEC conditions and the control-referencing does increase the +FF vs –FF symmetry compared to Figures 3 and S3, though not as completely as in Figures 4 and S4.

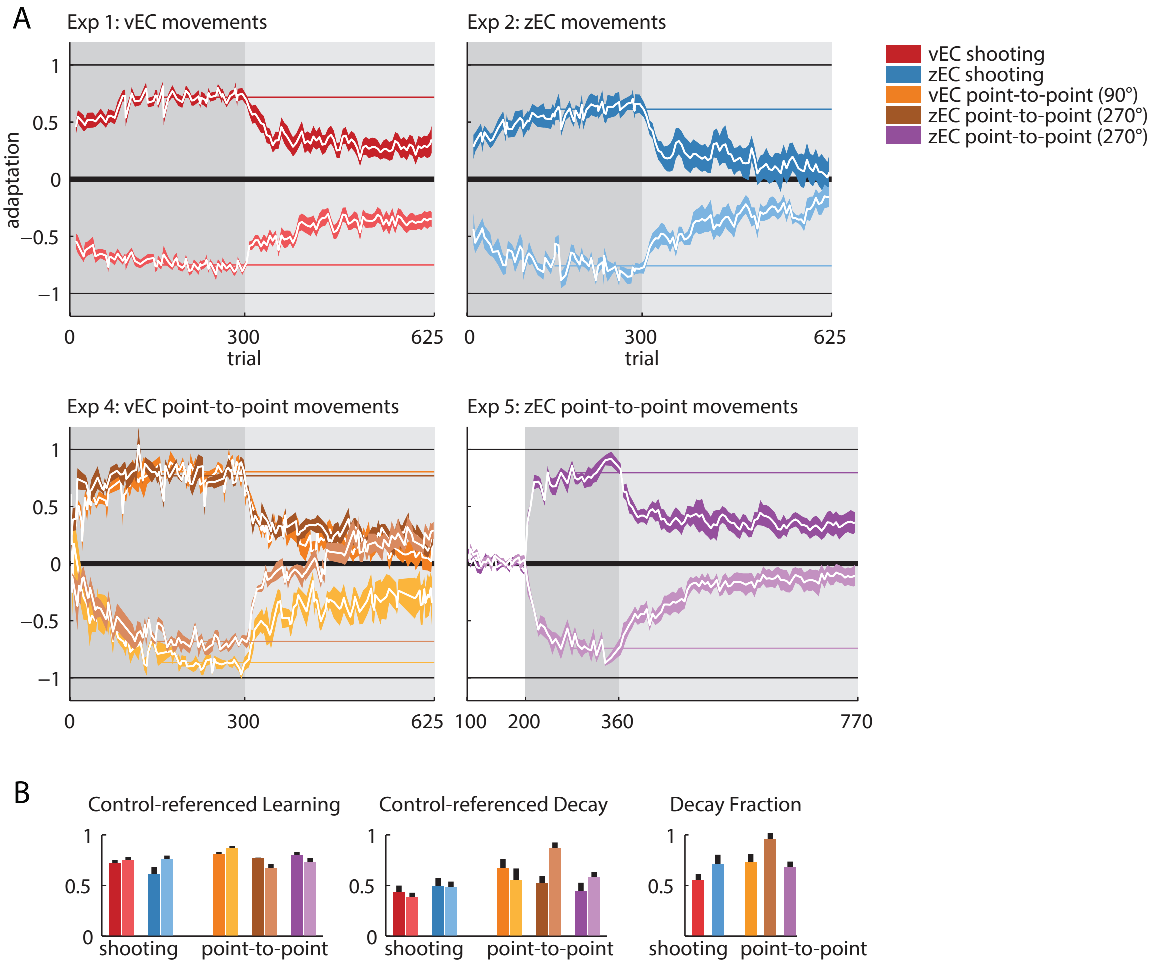

Figure S5: Learning and decay referenced to control data but using the adaptation coefficient to quantify adaptation. (A) Learning and decay for shooting movement experiments 1-2 and point-to-point movement experiments 4-5 after control-referencing and quantifying adaptation using the adaptation coefficient measure. **(B)** Amount of learning, amount of decay, and decay fraction for this control-referenced adaptation quantified using the adaptation coefficient measure. Learning and decay were quantified in the same manner as in Figure 3. These results are nearly as symmetric as the control-referenced Figures 4 and S4, which use integrated lateral force to quantify adaptation, and are much more symmetric than Figures 3 and S3, which are not control-referenced and use the same adaptation coefficient measure.
