# Supplementary material for: The Decay of Motor Memories Is Independent of Context Change Detection
Source: PLoS Comput Biol. 2015 Jun 25;11(6):e1004278. doi: 10.1371/journal.pcbi.1004278 (PMC4482542; doi:10.1371/journal.pcbi.1004278)
Supplement: S6 File — (PDF) [file pcbi.1004278.s006.pdf]

S6: Individual-level analysis of point-to-point movements indicates essentially no systematic delay in decay onset

We examined the delay in the individual participant point-to-point data in the same way as in the shooting data, by fitting exponential functions with unconstrained delay parameters to the error clamp trials during the last 150 trials of the learning period (trials -150 to 0) and the entirety of the retention period (trials 1-325 in experiment 4 and trials 1-410 in experiment 5).

We found similar results for the point-to-point movement data as for the shooting movement data, with near-zero median delays and no difference between the vEC and zEC conditions (medians of experiments 4 (90° movements), 4 (270° movements), and 5 (270° movements) are -3.0 (p=0.432), -6.5 (p=0.084), and 1.7 (p=0.625) respectively, p>0.1 for E4 (270°) vs E5 (270°)).

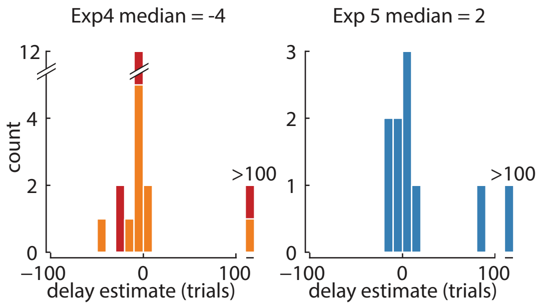

Figure S6: Individual-level analysis of decay onset time. Delay parameter estimates for experiments 4 and 5 are centered near zero with some positive and some negative estimated delays. The vEC condition (experiment 4) does not show more delay than the zEC condition (experiment 5), despite the masked context change that should make this change take longer to detect. These results are consistent with the shooting movements in Figure 6.
